# Connectivity-Guided Theta Burst Transcranial Magnetic Stimulation Versus Repetitive Transcranial Magnetic Stimulation for Treatment-Resistant Moderate to Severe Depression: Magnetic Resonance Imaging Protocol and SARS-CoV-2–Induced Changes for a Randomized Double-blind Controlled Trial

**DOI:** 10.2196/31925

**Published:** 2022-01-20

**Authors:** Stefan Pszczolkowski, William J Cottam, Paul M Briley, Sarina J Iwabuchi, Catherine Kaylor-Hughes, Abdulrhman Shalabi, Ben Babourina-Brooks, Adam Berrington, Shaun Barber, Ana Suazo Di Paola, Andrew Blamire, R Hamish McAllister-Williams, Jehill Parikh, Mohamed Abdelghani, Lars Matthäus, Ralf Hauffe, Peter Liddle, Dorothee P Auer, Richard Morriss

**Affiliations:** 1 NIHR Nottingham Biomedical Research Centre University of Nottingham Nottingham United Kingdom; 2 Mental Health and Clinical Neurosciences School of Medicine University of Nottingham Nottingham United Kingdom; 3 Sir Peter Mansfield Imaging Centre University of Nottingham Nottingham United Kingdom; 4 Institute of Mental Health School of Medicine University of Nottingham Nottingham United Kingdom; 5 Nottinghamshire Healthcare NHS Foundation Trust Nottingham United Kingdom; 6 Faculty of Medicine University of Jeddah Jeddah Saudi Arabia; 7 Leicester Clinical Trials Unit University of Leicester Leicester United Kingdom; 8 Translational and Clinical Research Institute Faculty of Medical Sciences Newcastle University Newcastle upon Tyne United Kingdom; 9 Cumbria, Northumberland, Tyne and Wear NHS Foundation Trust Newcastle upon Tyne United Kingdom; 10 Camden and Islington NHS Foundation Trust London United Kingdom; 11 eemagine Medical Imaging Solutions GmbH Berlin Germany; 12 NIHR MindTech MedTech and in Vitro Centre Nottingham United Kingdom; 13 NIHR Applied Research Collaboration East Midlands Nottingham United Kingdom

**Keywords:** depression, magnetic resonance imaging, image-guidance, personalized medicine, transcranial magnetic stimulation

## Abstract

**Background:**

Depression is a substantial health and economic burden. In approximately one-third of patients, depression is resistant to first-line treatment; therefore, it is essential to find alternative treatments. Transcranial magnetic stimulation (TMS) is a neuromodulatory treatment involving the application of magnetic pulses to the brain that is approved in the United Kingdom and the United States in treatment-resistant depression. This trial aims to compare the clinical effectiveness, cost-effectiveness, and mechanism of action of standard treatment repetitive TMS (rTMS) targeted at the F3 electroencephalogram site with a newer treatment—a type of TMS called theta burst stimulation (TBS) targeted based on measures of functional brain connectivity. This protocol outlines brain imaging acquisition and analysis for the Brain Imaging Guided Transcranial Magnetic Stimulation in Depression (BRIGhTMIND) study trial that is used to create personalized TMS targets and answer the proposed mechanistic hypotheses.

**Objective:**

The aims of the imaging arm of the BRIGhTMIND study are to identify functional and neurochemical brain signatures indexing the treatment mechanisms of rTMS and connectivity-guided intermittent theta burst TMS and to identify imaging-based markers predicting response to treatment.

**Methods:**

The study is a randomized double-blind controlled trial with 1:1 allocation to either 20 sessions of TBS or standard rTMS. Multimodal magnetic resonance imaging (MRI) is acquired for each participant at baseline (before TMS treatment) with T1-weighted and task-free functional MRI during rest used to estimate TMS targets. For participants enrolled in the mechanistic substudy, additional diffusion-weighted sequences are acquired at baseline and at posttreatment follow-up 16 weeks after treatment randomization. Core data sets of T1-weighted and task-free functional MRI during rest are acquired for all participants and are used to estimate TMS targets. Additional sequences of arterial spin labeling, magnetic resonance spectroscopy, and diffusion-weighted images are acquired depending on the recruitment site for mechanistic evaluation. Standard rTMS treatment is targeted at the F3 electrode site over the left dorsolateral prefrontal cortex, whereas TBS treatment is guided using the coordinate of peak effective connectivity from the right anterior insula to the left dorsolateral prefrontal cortex. Both treatment targets benefit from the level of MRI guidance, but only TBS is provided with precision targeting based on functional brain connectivity.

**Results:**

Recruitment began in January 2019 and is ongoing. Data collection is expected to continue until January 2023.

**Conclusions:**

This trial will determine the impact of precision MRI guidance on rTMS treatment and assess the neural mechanisms underlying this treatment in treatment-resistant depressed patients.

**Trial Registration:**

ISRCTN Registry ISRCTN19674644; https://www.isrctn.com/ISRCTN19674644

**International Registered Report Identifier (IRRID):**

DERR1-10.2196/31925

## Introduction

### Background

Major depressive disorder is the most disabling health condition in terms of years lived with disability [1] and has a life prevalence of approximately 13% of the general population [2]. Although antidepressants and psychotherapy are effective treatments for major depressive disorder, a number of patients do not respond to trials with ≥2 antidepressants [3,4]. These patients are categorized as patients with *treatment-resistant depression* (TRD), which is associated with increased rates of suicide, hospitalization, poor physical health, and increased health care costs [5].

Neuromodulation in TRD by means of transcranial magnetic stimulation (TMS) is cost-effective in comparison with standard care [6]. This approach attempts to directly modulate localized brain activity, by using an electromagnetic coil to target magnetic pulses to a specific region that in turn induce an electric current. Depending on the pattern of stimulation, this stimulation can modulate brain activity in either an excitatory or inhibitory manner [7]. The therapeutically desired activity modulation of specific brain regions also requires precise localization of the stimulation and understanding of its networks especially where the therapeutic target cannot be directly stimulated. It is also important to ensure that the selected brain region for stimulation is being stimulated by correctly determining its skull projections. This is currently done by using tape measures or electroencephalography caps. However, these measurements are prone to human error or differences in cap placement between sessions. Importantly, the variability in brain and skull anatomy of a population means that such *one-size-fits-all* targeting approaches are not appropriate in the context of precision neuromodulation. In addition to such anatomical variation, advances in brain connectomics highlight substantial interindividual variability of functional brain networks that is expected to have a direct impact of network-mediated downstream effects from superficial TMS. There is also a potential for target misalignment across multiple treatment sessions. Therefore, it is important to find alternative procedures to TMS treatment target estimation that are reproducible and to also consider precision-based approaches.

Neuroimaging studies in depression have consistently demonstrated altered connectivity within, and between, canonical resting-state networks, such as the default mode, salience, and central executive networks, adding to our current understanding of the dysfunctional brain circuitry involved [8-11]. Importantly, the dorsolateral prefrontal cortex (DLPFC) and insula influence each other in a reciprocal fashion and are key hubs of the central executive and salience networks, respectively, meaning that dysfunction of such a loop would have greater effects on the networks. The United States Food and Drug Administration and National Institute of Health and Clinical Excellence approved TMS treatments in TRD target the left DLPFC, which likely exerts its treatment effect by modulating these deeper, connected brain regions and networks [12,13]. Importantly, to take into account the known interindividual variability in the tissue cytoarchitecture [14], and more pertinently, the functional connectivity (FC) of the DLPFC [15], individualized TMS targeting may be important for the efficacious delivery of treatment [13,16]. In addition, studies have shown that FC of the DLPFC can predict the clinical efficacy of TMS [13,17], that reproducible individual TMS targets can be created [16], and that treatment using this individualized targeting can improve the response rate above those reported in a recent meta-analysis of repetitive TMS (rTMS) treatments from approximately 45% to 50% to approximately 64% [18,19]. Recent studies provide further evidence of the strength of personalized TMS treatment targeting, with one nonrandomized trial observing an 86% remission rate in response to personalized and accelerated intermittent theta burst stimulation (TBS) treatment [20]. Further support for the importance of personalized neuromodulation comes from a small randomized controlled trial that shows improved efficacy the nearer the standard rTMS target was to an in silico determined connectivity-based personalized target [21]. In addition to creating personalized treatment targets for TMS, the use of neuroimaging in longitudinal studies of TMS provides an opportunity to both mechanistically evaluate the treatment effect on the intended treatment targets and to assess response prediction. A recent large-scale imaging study collated >1000 depressed participants’ resting-state functional magnetic resonance imaging (rsfMRI) and using a data-driven canonical correlation analysis reported a biotype characterized by the connectivity between the insula and other brain regions that significantly predicted treatment response to TMS and was a stronger predictor of response than clinical measures alone [17]. Smaller studies have also provided evidence that baseline FC with the DLPFC can predict clinical efficacy [13,18]. Although there is growing evidence that brain connectivity patterns can predict treatment response, inconsistencies in the data demonstrate a clear need for further prospective investigation in a large, well-characterized sample.

The Brain Imaging Guided Transcranial Magnetic Stimulation in Depression (BRIGhTMIND) study [22] is a randomized, double-blind controlled trial comparing the cost-effectiveness, efficacy, and mechanistic effects of 2 neuromodulation approaches in TRD—standard the US Food and Drug Administration–approved rTMS [23] and connectivity-guided intermittent theta burst TMS (cgiTBS). Theta burst TMS corresponds to an alternative patterned form of standard rTMS that uses high (gamma) frequency pulses, repeating at lower (theta) frequency intervals. In this study, both standard rTMS and cgiTBS treatment locations on the head are determined in a repeatable and personalized manner based on magnetic resonance imaging (MRI) images obtained. However, the standard rTMS location is based only on the overall shape of the head, whereas the cgiTBS location is determined by the head shape, brain anatomy, and FC profile of the participant. Each participant is randomized at the first TMS session to either 20 sessions of rTMS or 20 sessions of cgiTBS performed daily for 4 to 6 weeks. Clinical and economic outcomes, including the primary outcome measure—the 17-item Hamilton Depression Rating Scale (HDRS-17) [24], are assessed by blinded research assessors at baseline, 8 weeks, 16 weeks, and 26 weeks. Clinical data are analyzed on an intention-to-treat basis. Further details of the clinical protocol have been published [22] and can be found in the clinical trials register (ISRCTN19674644). This paper describes the SARS-CoV-2–related changes to the study, including revised recruitment target, outcome measure and power calculation, MRI protocol, TMS target generation, interim baseline analyses, and mechanistic imaging outcomes for the BRIGhTMIND study.

### Primary Objectives

The objectives of the imaging arm of the BRIGhTMIND study are to identify functional and neurochemical brain signatures indexing the treatment mechanisms of rTMS and cgiTBS and to identify imaging-based markers predicting response to treatment.

#### Primary Hypotheses for Mechanistic Imaging Study

The primary hypotheses for the mechanistic imaging study are as follows:

1. Treatment response, as measured using change in the HDRS-17, will correlate with posttreatment changes in DLPFC-dorsomedial prefrontal cortex (DMPFC) FC at 16 weeks.
2. Treatment response (HDRS-17) to TMS treatment can be predicted using baseline insula-DLPFC effective connectivity.
3. Connectivity-guided intermittent TBS treatment-related γ-aminobutyric acid (GABA) changes will be correlated with a reduction in HDRS-17 at 16 weeks.

#### Exploratory Aims for the Mechanistic Imaging Study

The exploratory aims for the mechanistic imaging study are as follows:

1. To identify imaging-based biotypes that predict treatment response in TRD patients
2. To further study the neural mechanisms underlying therapeutic efficacy by assessing interrelations of changes in complex brain network metrics with improvement of clinical symptoms
3. To determine the distance between the applied rTMS and TBS treatment targets with an in silico subgenual anterior cingulate cortex (sgACC) seeded DLPFC target, and its association with therapeutic efficacy (change in HDRS-17 at 16 weeks)

### Interim Analyses of Baseline Data

Analysis of baseline MRI and clinical data will explore seven themes that will support and boost both the impact and inference of the main study outcomes. These themes are (1) treatment resistance, (2) comorbid anxiety, (3) cognitive impairment, (4) trauma, (5) medication and other confounds, (6) interlinking analyses, and (7) model building. Themes 1-5 will be driven by the testing of distinct hypotheses based on previous knowledge and literature. Themes 6 and 7 are exploratory as they are provided to allow expansion of the proposed analyses into further tests in different MRI modalities and measures, and to use any resulting findings from all the above themes into building a model-based brain signature of TRD. Given the limited literature published on TRD and the rich data set available from the BRIGhTMIND study, it is important that there is room to carry out both hypothesis-driven and exploratory analyses. All baseline analyses will remain blinded to treatment allocation (cgiTBS or rTMS) and trial outcomes (responder or nonresponder) until after the final database lock. The specifics of these planned analyses can be found in the study by Cottam et al [25].

## Methods

### Ethics Approval

Ethical approval was granted by East Midlands Leicester Central Research Ethics Committee (ref: 18/EM/0232) on 30 August 2018.

### Recruitment and Imaging Sites

In the initial setting, there will be 4 recruitment sites from across the United Kingdom National Health Services (NHS) (Nottinghamshire Healthcare Foundation NHS Trust, Nottingham; Northamptonshire Healthcare NHS Foundation Trust, Northampton; Cumbria, Northumberland, Tyne and Wear NHS Foundation Trust, Newcastle; and Camden and Islington NHS Foundation Trust, London), which will deliver the TMS treatments locally. However, only 3 sites will perform imaging, as all Northampton participants will be scanned at the Nottingham site. The possibility of incorporating additional sites (either for recruitment and scanning or for recruitment only) will be kept open throughout the course of the BRIGhTMIND study and will be evaluated case by case, based on achieved recruitment. However, Northampton did not reopen to recruitment in August 2020 and was informed of the decision to close the site in December 2020 owing to low recruitment numbers. A new site is being opened in Oldham (Pennine-Care NHS Foundation Trust) in the summer of 2021 to increase recruitment. Both scanning and TMS treatments will be delivered locally at the Oldham site.

### MRI Acquisition

MRI scans are acquired at 2 time points. First, a baseline scan is acquired within 14 days of the baseline assessment, after which treatment randomization occurs. Allocated TMS treatment is then initiated within 14 days of the MRI scan. The second MRI scan is acquired within 14 days of the 16 weeks follow-up assessment [22]. Both MRI scans will be carried out at the same site and using the same scanner platform, with the same list of images acquired at each time point.

The participants will undergo multimodal MRI at 3T. We will acquire a core protocol across all treatment sites consisting of a structural T1-weighted scan and an eyes-open blood oxygenation level dependent (BOLD) echo-planar imaging (EPI) rsfMRI scan with additional positive and negative phase-encoded images to enable distortion correction. For the mechanistic substudy, all sites will also acquire a diffusion-weighted scan (with an additional negative phase-encoded B0 image for distortion correction), whereas 2 sites (Nottingham and Northampton) will also acquire an arterial spin labeling (ASL) scan, and 3 sites (Nottingham, Newcastle, and Northampton) will acquire a Meshcher-Garwood Point Resolved Spectroscopy (MEGA-PRESS) magnetic resonance spectroscopy (MRS) scan. General descriptions of the scanning sequences are delineated in the next paragraph, with further details available in Multimedia Appendix 1. A total of six scanners are used within the BRIGhTMIND study, and the details are as follows: Newcastle—Achieva dStream (Philips); London—Prisma (Siemens); Oldham—Achieva (Philips); Nottingham and Northampton will use the following three scanners (sequentially) over the course of the trial owing to a scanner upgrade carried out midtrial: (1) Discovery MR750 (GE Healthcare); (2) Ingenia (Philips); and (3) Premier (GE Healthcare). All sites are instructed to use 32-channel head coils for the study, and a 48-channel head coil will be used at the GE Healthcare Premier.

**Figure 1:**
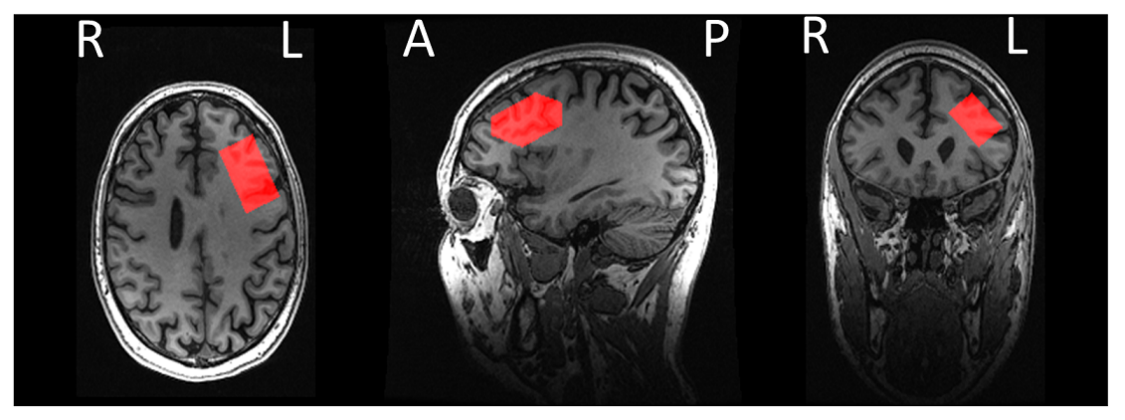
An example of the dorsolateral prefrontal cortex magnetic resonance spectroscopy voxel placement. A: anterior; L: left; P: posterior; R: right.

High-resolution T1-weighted images will be acquired using sagittal fast-spoiled gradient echo BRAVO (or equivalent) sequences with 1 mm^3^ isotropic voxels covering the whole head from the vertex to the neck. rsfMRI images will be acquired with the eyes open using a fixation cross. All sites are instructed to use a gradient echo EPI sequence aligned with the anterior commissure-posterior commissure line, with acquisition covering from the vertex downward (repetition time [TR]/echo time [TE]=2000/32 ms; flip angle=77°; 35 slices; voxel size=3 mm^3^; slice gap=0.5 mm; field of view=192×192 mm; interleaved bottom/up; 240 volumes; phase encoding direction=posterior>anterior). All rsfMRI images will have associated forward- and reverse-phase-encoded B0 images acquired to facilitate distortion correction. Importantly, these images will be acquired with the same image dimensions as the rsfMRI and will be acquired directly before the rsfMRI. Diffusion-weighted images will be acquired and aligned with the anterior commissure-posterior commissure line, with coverage beginning at the vertex and extending inferiorly (TR/TE=11,000/minimum ms; flip angle=90°; 55 slices; voxel size=2 mm^3^; field of view=220×220 mm; 64 directions [B=1000]; 5 B0 images; phase encoding direction=anterior>posterior; sense factor=0). In addition, a reverse phase-encoded B0 image (posterior>anterior) will also be collected with all other parameters and coverage matched with the diffusion-weighted image acquisition to enable distortion correction. ASL on the Discovery MR750 (GE Healthcare) will be carried out using the vendor-supplied 3D-pseudocontinuous ASL sequence with whole-head coverage, and the participants will be instructed to keep their eyes open (TR/TE=4632/10.5 ms; inversion time [TI]=2025 ms; flip angle=111°; 36 axial slices; voxel size=1.875×1.875×4 mm; field of view=240×240 mm). Further ASL sequences on the Ingenia (Philips) and Premier (GE Healthcare) will be set up to replicate the original acquisition (see Multimedia Appendix 1 for details). MRS will be acquired via a voxel in the left DLPFC, which is placed to the best match as shown in Figure 1. The acquisition at both the Newcastle and Nottingham scan sites will be a MEGA-PRESS GABA editing sequence (voxel dimensions are 45×30×20 mm for anterior/posterior [A/P], left/right [L/R], inferior/superior [I/S] directions, respectively; TR/TE=2000/68 ms; 320 averages) acquired using the schema described by Mikelsen et al [26].

### Image Archiving and Quality Control

Subject digital imaging and communications in medicine (DICOM) session files are uploaded onto an XNAT (Washington University School of Medicine) [27] database infrastructure for all data other than MRS data using anonymized subject numbers. Once the session is archived within XNAT, it is put into a quarantined state awaiting quality control (QC), and DICOM files are automatically converted into Brain Imaging Data Structure [28] NIFTI or JSON pairs for each scan using the dcm2bids-session v1.5 XNAT container, with T1-weighted images also undergoing defacing within this step. Defacing consists of the registration and application of a mask that removes the lower portion of the nose and the mouth and jaw to the T1-weighted image. Thus, allowing the nasion and ears to be used for TMS target generation. Then, the T1-weighted and BOLD rsfMRI scans are manually assessed using the MRIQC v0.11.0 [29] QC XNAT container. If both the T1-weighted and BOLD images within a session pass QC, then the session is removed from the quarantined state. T1-weighted image reports from MRIQC are assessed for and judged to pass QC if there is full head coverage and no visual artifacts or incidental findings. BOLD data MRIQC reports are checked for frame-wise displacements larger than 3 mm, average frame-wise displacement over 1 mm, image artifacts, or long-lasting intensity changes owing to motion in the carpet plot. If any of these are present, then the BOLD image fails QC. Note that only nonquarantined data are subsequently downloaded from the XNAT database for preprocessing and further analysis.

The MRS data are handled differently from the rest of the imaging data, as it is uploaded separately onto XNAT for participants whose structural T1-weighted and rsfMRI data have passed QC only. The QC for MRS data is carried out during preprocessing and is described later in this paper.

### T1-Weighted and BOLD Image Preprocessing

Both structural T1-weighted and BOLD rsfMRI data are preprocessed with the SPMIC-BRC pipeline v1.2.6 [30,31]. This pipeline is based on tools from the following packages: Statistical Parametric Mapping v12 (SPM12 [32]), FMRIB Software Library (FSL) v5.0.11 [33], and Freesurfer v6 [34].

Structural T1-weighted images are first coarsely brain extracted using the FSL brain extraction tool (BET) [35] and their field of view is reduced by removing the lower head and neck using FSL *robustfov*. The original and brain extracted images are then nonlinearly registered to the MNI152 1-mm template [36] using FSL FMRIB’s nonlinear image registration tool (FNIRT). The original FSL BET brain extraction is then refined by applying the produced nonlinear transformation to warp the MNI152 brain mask onto the subject’s T1 image [37-39]. The resulting brain extracted image is finally bias-corrected and segmented into gray matter, cerebrospinal fluid (CSF), and white matter (WM) using FSL FMRIB Automated Segmentation Tool (FAST) [40]. The resulting WM and CSF probability maps are binarized at a tissue-probability threshold of 98% and then eroded using a spherical kernel with a radius of 2 voxels.

The BOLD rsfMRI images undergo EPI distortion correction by inputting the positive and negative phase-encoded acquisitions into TOPUP [41]. Then, they undergo between-volume motion correction (MCFLIRT 6DoF) and SPM12 interleaved slice-timing correction (bottom-up) [38]. The corrected BOLD image is subsequently smoothed with a 5 mm full-width half-maximum kernel using Smallest Univalue Segment Assimilating Nucleus (SUSAN) [42] and denoised with ICA-AROMA [43]. BOLD images are high-pass filtered at a frequency of 0.01 Hz after denoising. A transformation between the resulting BOLD image and the T1-weighted image is later computed using FSL *epi_reg*, and then combined with the TOPUP spatial distortion correction transformation. The resulting combined transformation is then inverted to create a nonlinear transformation from the T1-weighted to (original uncorrected) BOLD space. The previously computed binary WM and CSF masks are later warped into BOLD space using the T1-weighted to BOLD transformation to extract the WM and CSF time series from the BOLD data. To control for additional physiological or scanner-related noise, the WM and CSF time series are then regressed out of the rsfMRI time series.

Although the above pipeline will remain locked during the trial for the calculation of TMS target coordinates, any analyses carried out for final publication will seek to use further developed state-of-the-art pipelines to make the best use of the data at the time. Thus, later versions of software or new tools may be incorporated into this pipeline and those detailed below.

### MRS Preprocessing

#### MRS Processing

The MRS data will be processed using an in-house routine developed in MATLAB (MathWorks Inc) before metabolite quantification. All routines used will be made available to other researchers at the end of the trial via GitHub.

The GE Healthcare (P-file) MRS data will be coil-combined using the phase and maximum amplitude of the acquired unsuppressed water reference. Philips (.sdat or .spar) data are already coil-combined by the vendor software. Data will be split into 160 OFF and 160 ON spectra and eddy current corrected using the internal water reference. To minimize artifacts caused by subject motion or frequency drift, each spectrum will be frequency-and phase-corrected to the mean OFF spectrum using spectral registration [44,45]. Spectra with mean square error >3 SDs over the choline peak are automatically rejected as outliers [44,45]. The aligned ON and OFF spectra will subsequently be averaged to the 1 ON and 1 OFF spectra. The ON and OFF spectra will be aligned and subtracted to create the ON-OFF (difference) spectrum.

#### Metabolite Quantification

Unedited OFF spectra will be analyzed between 0.2 ppm and 4.0 ppm with linear combination model (LCModel; version 6.3-1H) [46,47]. Sequence-specific basis sets will be generated for each implementation of MEGA-PRESS in the study using density matrix simulations considering interpulse timings and radiofrequency pulses [48]. The OFF spectra will be used to quantify total *N*-acetylaspartate (tNAA=NAA+*N*-acetyl aspartylglutamate), total creatine (tCr=Cr+phosphocreatine), total choline (tCho=glycerophosphoryl choline+phosphorylcholine), mIns (*myo*-inositol), Glx (glutamate and glutamine), and taurine (Tau) levels. The edited ON-OFF MEGA-PRESS spectra will be analyzed in the range of 0.2-4 ppm with LCModel, using sequence-specific basis sets simulated using the same techniques as outlined for the OFF spectra.

Metabolite concentration values will be reported as ratios relative to tCr and Glx. In addition, water-scaled values, relative to the unsuppressed water signal, will be reported. For the latter, corresponding structural T1-weighted images will be segmented via SPM12 [49] into gray matter, WM, and CSF and aligned with the MRS volume using an in-house software. Water-scaled metabolite values will be corrected for partial volume effects and T1/T2 relaxation in accordance with the procedure by Gasparovic et al [50].

Initial QC criteria will be applied to reject data based on the raw spectral linewidth of the unsuppressed water<13 Hz [51] and also the signal-to-noise ratio of OFF spectra 3 SD below the mean for the study in line with recent consensus recommendations. The spectra will also be visually inspected for lipid, macromolecule, and subtraction artifacts by an MRS expert.

### Identification of TMS Targets

#### cgiTBS Treatment Target

Once the BOLD rsfMRI sequence is preprocessed, an independent component analysis is performed on its volumes using MELODIC v3.15 [52]. The resulting z-scored components are filtered by zeroing values <1.96, and the component most correlated with a left central executive network (lCEN) *Z*-score map (derived from the lCEN network shared by Smith et al [53]; [component 13/20] thresholded at *Z-*score >1.96, and then manually masked to include only the cluster in the frontal gyri) is found. This most correlated component is subsequently masked with a left middle frontal gyrus (lMFG) region of interest (derived from the middle frontal gyrus from the Harvard-Oxford cortical atlas [54-57] thresholded at >35% probability to exclude the precentral gyrus, and secondarily masked to exclude voxels in the precentral gyrus and right hemisphere). Both the lCEN map and lMFG mask in the BOLD space are computed from the MNI152 space using a warp, which is the result of concatenating the MNI152 to T1-weighted and the T1-weighted to BOLD space transformations. Preprocessed BOLD images that have been previously fed into MELODIC are then fed into a bivariate first-order coefficient-based voxel-wise Granger causality analysis (GCA) using the rsfMRI Data Analysis Toolkit v1.8 toolbox [58] in MATLAB 2014a. GCA is used to compute the effective connectivity from the right anterior insula (rAI; 6-mm sphere centered on MNI voxel coordinates x=30, y=24, z=−14 from McGrath et al [59]) to the area within the MFG region of interest defined previously, as described by Iwabuchi et al [18]. Specifically, only the X-Y (rAI to lCEN) output is used to find the connectivity peak. The rAI mask in the BOLD space is computed in the same manner as the lCEN and lMFG masks. Finally, the cgiTBS brain target is defined as the peak of the most significant Z-score GCA cluster.

#### rTMS Treatment Target

The rTMS brain target is determined by taking the scalp F3 voxel coordinate in the MNI152 space defined in Tsuzuki et al [60] (x=−49.0, y=51.0 mm, z=40.0 mm) and computing the voxel in the brain parenchyma that is closest to it (ie, x=−41.0, y=43.0 mm, z=32.0 mm). This brain voxel is then nonlinearly projected into BOLD space by using a combination of the MNI152 to T1-weighted and T1-weighted to BOLD transformations.

#### Target Generation

**Figure 2:**
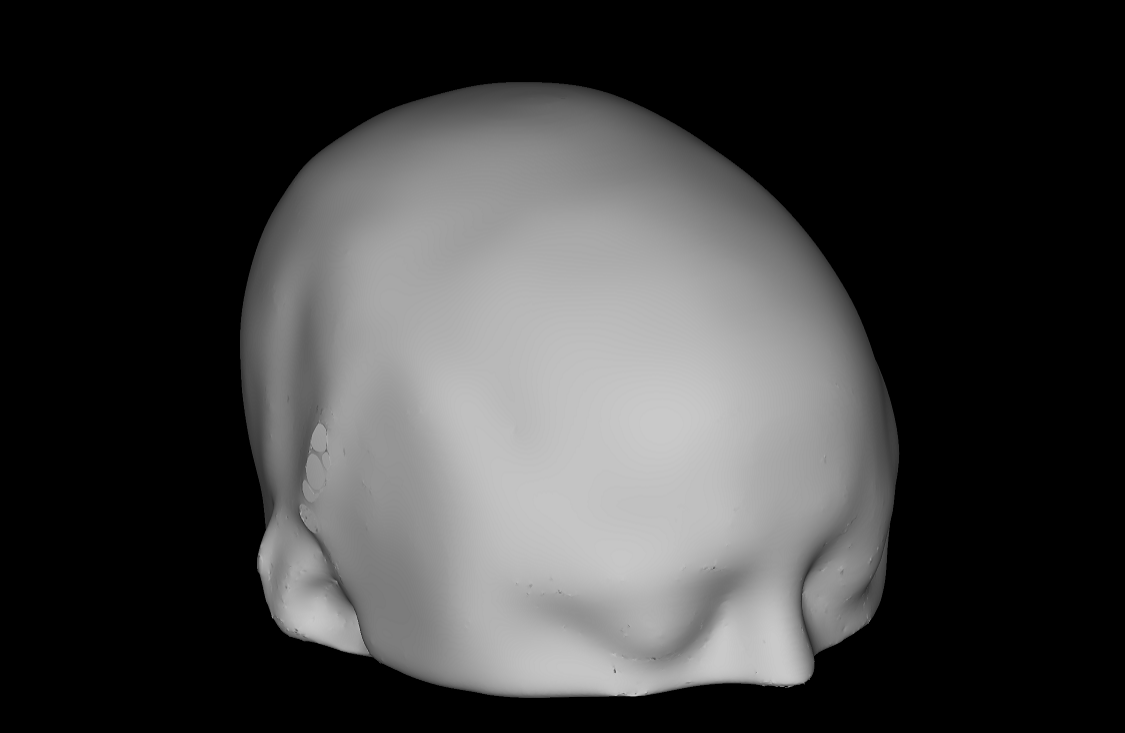
An example of a generated head mesh.

To ensure that TMS treatment is administered as close as possible to the brain target point (computed as either a cgiTBS or rTMS target), the scalp projection and angulation of the TMS wand is computed. The MNI152 brain mask is first transformed into the subject T1-weighted space and then all the background voxels in the T1-weighted image are set to zero. Background voxels are considered to be voxels that are outside the transformed brain mask or have an intensity value below a certain threshold set by visual inspection. A 3D mesh model of the head is then created using Freesurfer *mkheadsurf* script with the processed T1-weighted image (converted into Freesurfer MGZ format) as input and 100 smoothing steps, and then transformed into T1-weighted space using the *tkr2scanner* transformation matrix of the MGZ file. Note that, as the lower head and neck are previously removed from the T1-weighted image, the resulting head model does not include enough facial features (such as the lips or chin) to uniquely identify the participant. Therefore, their anonymity is preserved (Figure 2).

**Figure 3:**
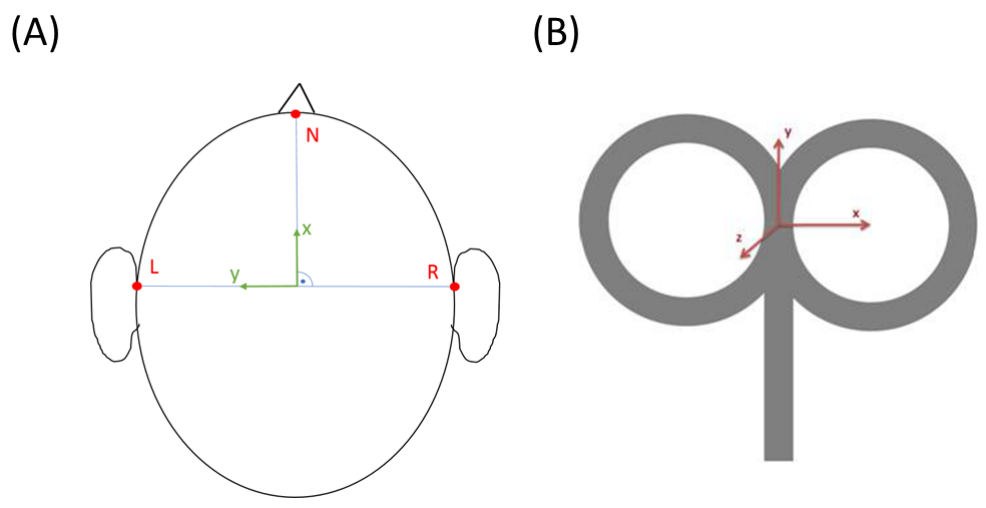
Nasion-left-right coordinate system (a) and coil coordinate system (b).

In the following stage, the nasion and both the left and right preauricular coordinates are manually annotated on the T1-weighted image. Then, the nasion and preauricular points on the head 3D mesh are defined as the vertices closest to the manually determined coordinates. This allows the construction of a nasion-left-right (NLR) coordinate system for the head mesh model, which is measured using the same units as the T1-weighted space (ie, mm). The idea behind the use of this coordinate system is that it is invariant to head positioning. The origin of the NLR coordinate system lies on the intersection between the line that passes through both preauricular points and the perpendicular line that passes through the nasion. The x-axis points toward the nasion, the y-axis points toward the left preauricular point, and the z-axis points toward the top of the head (Figure 3A). In order to convert coordinates in T1-weighted space into NLR space, the coordinates of the NLR origin in T1-weighted space are computed and the coordinates of the origin, nasion and preauricular points in NLR space are subsequently calculated as follows:




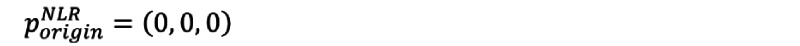




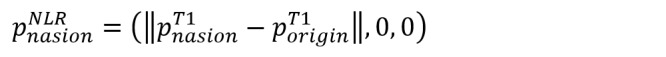




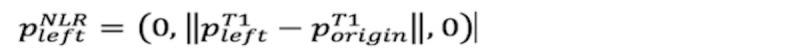




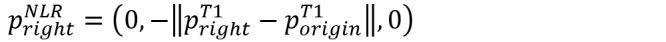




**Figure 4:**
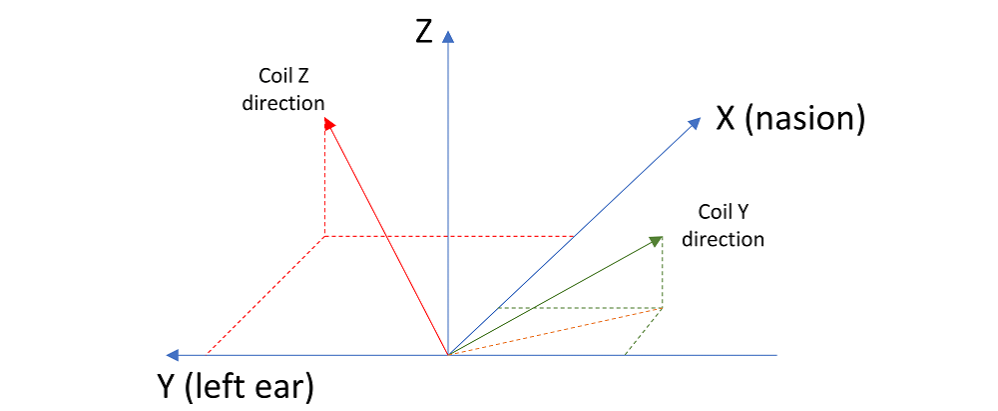
Transcranial magnetic stimulation wand positioning rule with respect to the nasion-left-right (NLR) coordinate system. The z-axis of the coil (in red) should be normal to the treatment target on the head mesh. The projection (in orange) of the y-axis of the coil (in green) into the NLR xy-plane forms a 45° angle with the midsagittal plane (NLR x-axis).

Here, 
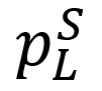
 correspond to the coordinates of landmark *L* in *S* space, and ‖∙‖ is the L2 vector norm. Finally, given the 2 sets of 4 points, the optimal rigid transformation that maps T1-weighted space points into their NLR coordinates is computed using a Least-Squares approach [19,61]. This rigid transformation is then used to transform the cgiTBS and rTMS brain targets into NLR space. The treatment targets in the head mesh are then computed as the mesh point closest to its corresponding brain target. Finally, to determine the angulation of the TMS treatment coil, a coil coordinate system is created. In this coordinate system, the x-axis points toward the right of the wand, the y-axis points toward the front and the z-axis points upward (Figure 3B). The wand positioning must be such that the z-axis of the coil is normal to the head mesh at the target mesh vertex, and the projection of the y-axis of the coil onto the NLR xy-plane must form a 45° angle with the midsagittal plane (ie, the NLR x-axis), as depicted in Figure 4. Finally, to compute the rotation matrix that transforms coil coordinates into NLR coordinates, a procedure analogous to the one that transforms T1-weighted space coordinates into NLR coordinates is followed.

Treatment delivery on the BRIGhTMIND study will be performed using a 70-mm figure-of-eight coil (Ez Cool coil) and a Magstim Horizon Performance Stimulator with StimGuide Navigated TMS Package (Magstim Company). Therefore, the final step in the process is to generate a StimGuide compatible file with the computed treatment targets and rotation matrices in quaternion form [62]. The last QC step before sharing these calculated targets with the appropriate recruitment site is to visually assess the scalp surface on which the TMS stimulation angle is calculated. Targets are recalculated only if the scalp surface around the treatment sites is too noisy, which appears visually as bumps on the surface. The image threshold given is then increased iteratively until the scalp surface appears smooth.

### Imaging Analyses on Mechanistic TMS Treatment Effects

#### Response Definition

Although the clinical trial is outlined in full in the former protocol paper [22], it is important to note that the clinical response within the BRIGhTMIND trial has been defined as a 3-point reduction in depression symptoms (averaged over all 3 posttreatment-time points at 8, 16, and 26 weeks, respectively) from baseline on the HDRS-17 [63]. All imaging tests outlined here will use this outcome as their variable for changes in depression symptoms.

#### Primary Mechanistic Outcomes

The primary mechanistic outcomes are as follows:

1. Correlation of change in depression symptoms following TMS with changes in DLPFC-DMPFC FC at 16 weeks (post minus pretreatment)
2. Correlation of change in depression symptoms following TMS with baseline effective connectivity (as calculated for the cgiTBS target) between the rAI seed region and the DLPFC (defined as a 6 mm spherical region of interest centered on the subject’s cgiTBS target coordinate)
3. Correlation of change in depression symptoms following TMS with cgiTBS treatment-related GABA changes (post minus pretreatment) at 16 weeks

#### Exploratory Outcomes

The exploratory outcomes are as follows:

1. Exploratory outcomes include, but are not limited to, evaluation of FC-based biotypes in the prediction of individual treatment response in TRD patients (25% change in HDRS-17 score from baseline to 16 weeks) [17] and to further study the neural mechanisms underlying therapeutic efficacy by assessing interrelations of changes in complex brain network metrics (including the use of graph analysis) with changes in clinical symptoms.
2. Exploratory outcomes include, but are not limited to, change in depression symptoms following TMS (HDRS-17 score post minus pretreatment) with baseline peak negative FC between the sgACC-left DLPFC and correlation of treatment response with distance between coordinates of the peak negative sgACC-left DLPFC FC and the calculated cgiTBS target.

### Data Analysis

Clinical, demographic, and psychometric data will be compared between groups and conditions using the latest version of appropriate statistical software such as SPSS (IBM Inc) or R (R Foundation).

Imaging data will be analyzed using the latest versions of established toolboxes where possible, but as the T1-weighted and rsfMRI preprocessing pipelines have been defined to calculate TMS stimulation targets, this preprocessed data will also be used for further statistical analysis where appropriate. Toolboxes will include (but are not limited to) the following: FSL, SPM12, rsfMRI Data Analysis Toolkit, Brain Connectivity Toolbox, and LCModel for MRS data. Analysis of ASL and diffusion MRI data will be described in subsequent manuscripts as they are not used to create treatment targets, nor are they required to test the stated mechanistic hypotheses.

### Study Amendments Because of SARS-CoV-2

Recruitment to the study was suspended on March 19, 2020, because of the SARS-CoV-2 pandemic. The study was reopened with a range of changes to protect participants and staff from SARS-CoV-2 on August 1, 2020, with recruitment at a slower permitted rate starting on September 1, 2020, at 3 of the 4 original study sites. The Northampton site was unable to restart, but another site at Oldham Greater Manchester has been opened to recruit for the study. Recruitment of the original sample of 368 participants was no longer possible within the resources and time-frame available.

In-person assessments were minimized owing to SARS-CoV-2 upon the study restart in August 2020, and as such, all assessments except the THINC-Integrated Tool (THINC-it; THINC-it Task Force [64]) task were carried out remotely either over the phone or via videoconference. THINC-it assessments were initially planned to take place at baseline and at the 8th, 16th, and 26th week in-person assessment. As these were no longer taking place in person, the THINC-it is now assessed only at baseline and 16 weeks, when the participant visits the center for their MRI scans. This was decided on to ensure that THINC-it assessments would remain consistent regarding both face-to-face instructions and the hardware used.

Using the expertise and independent advice of 2 independent committee monitoring and checking the progress of the study and in discussion with our funders who also sought external peer review, we changed the primary outcome measure from the binary variable of responder or nonresponder at 16 weeks (response was defined as a 50% drop or greater in the HDRS-17 from baseline to 16 weeks) to the mean change in the total score on the HDRS-17 across all follow-up time points (8, 16, and 26 weeks).

The National Institute of Clinical Excellence in its 2004 clinical guideline for depression defined a minimum clinically important difference in the total score on this scale of 3 points [65], which was reaffirmed in their 2009 guidelines [66]. We based our revised sample size on detecting an average difference of 3 points in the HDRS-17 at 8, 16, and 26 weeks, assuming an SD of 8 points based on our pilot study and a multicenter randomized controlled trial in chronic persistent depressive disorder led by the chief investigator [67], a correlation of 0.7 between the follow-up points (1 baseline measure with a correlation of 0.27 to the follow-up measures) and 20% loss to follow-up or drop-out, which was the average loss to follow-up across all follow-up points in the study as of January 31, 2021.

Under the assumptions mentioned above, a sample size of 266 participants will be required to achieve 89.3% power to detect the average difference of 3 points in the HDRS-17 at 8, 16, and 26 weeks at a 5% significance level (2-tailed); hence, 266 is the revised target for recruitment to the study. Recruitment of the sample will continue until January 31, 2022 and is limited by the resources available to the study. Given the uncertainties of recruitment to the study in the current pandemic, we note that under the same assumptions, a sample size of 232 would reassuringly still yield 85.1% power.

### Mechanistic Power Calculation for the Study

If 266 participants are randomized into the study, we expect 116 participants to provide full data for the mechanistic substudy (DLPFC-DMPFC FCp; change at 16 weeks and HDRS-17 change at 16 weeks), based on the observed acquisition rate to date (92/210, 43.8% provided data for both). The remaining 56.2% (118/210) without full mechanistic substudy data are because of a combination of participants being randomized at the London site (where only baseline scans are carried out), loss to follow-up at 16 weeks for HDRS-17 score, and loss to MRI follow-up. If we allow for 5% further loss owing to poor imaging quality, this will give us a total of 120 participants to assess the correlation of DLPFC-DMPFC FC change at 16 weeks and HDRS-17 change at 16 weeks.

A sample size of 120 achieves 96.3% power to detect a difference of −0.3 between the null hypothesis correlation of 0.2 and the alternative hypothesis correlation of 0.5 using a 2-sided hypothesis test with α=.05. The null hypothesis of 0.2 represents a very weak or weak correlation, whereas the alternative of 0.5 represents a moderate correlation [68]. The correlation between DLPFC-DMPFC FC change at 12 weeks and HDRS-17 change at 12 weeks in our pilot data was 0.58.

We must address 2 points regarding this aim. First, we previously had hypotheses 1 and 2 in reverse order, powering the substudy on the ability of the DLPFC-DMPFC FC change to discriminate between responders and nonresponders. The reason for moving away from this was because of the change in minimum clinically important difference criterion, such that by performing a correlation analysis, we assessed a noncategorized version of this test that removed the need to incorporate a response criterion but also improved statistical power owing to the use of the whole sample. Second, the proposed test assumes a weak correlation rather than no correlation. This approach was taken to make the test more robust against serial effects unrelated to treatment in the absence of a *no treatment* arm to the trial.

Regarding primary hypotheses 2 and 3, sensitivity analyses are presented to evidence that the revised sample size for the study will still allow for testing of these hypotheses. Sensitivity analysis based on a 1-tailed test with α=.05, 90% power, and a sample size of 232 for hypothesis 2 suggests a required effect size of |ρ|=0.19. This correlation of effective connectivity from the insula-DLPFC with changes in HDRS-17 at 12 weeks in our pilot data was −0.26. Sensitivity analysis following the same formula but with a lower expected sample size of 111 (as outlined for hypothesis 1) for hypothesis 3 suggests a required effect size of |ρ|=0.27. The correlation between GABA and HDRS-17 changes at 12 weeks in our pilot data was 0.68. Taken together, the study is expected to have the power to test the proposed hypotheses.

## Results

The first participant was recruited on January 22, 2019, but recruitment was suspended on April 23, 2020, because of the SARS-CoV-2 pandemic. Recruitment began again in August 2020 and is ongoing. To date, 194 participants have been randomized for treatment.

## Discussion

Neuromodulation via TMS has been shown to reduce the symptoms of TRD. However, using traditional identification of treatment locations, such as tape measurements or electroencephalography caps, may not be as effective as using personalized location estimation based on MRI. The BRIGhTMIND imaging substudy will assess the effectiveness and mechanistic effect of 2 variants of TMS, where treatment locations will be determined in a personalized manner using MRI. In addition, this study will evaluate whether treating a FC-based location is more effective than treatment based on a standard F3 location. If this hypothesis is found to be true, it may serve as evidence of the importance of using precision medicine in mental health.

An important point within an imaging trial such as this is the QC and amalgamation of data from different imaging scanners and locations. Differences in acquisitions, scanner hardware, and geographic locations can lead to problems when combining data for analysis. To tackle these potential variations, this study has put in place standardized image acquisition protocols, QC protocols, and data analysis pipelines with all data analysis occurring through the lead center at Nottingham. The use of automated QC tools such as MRIQC (T1-weighted and functional MRI) allows investigators to make clear decisions on whether the data are of acceptable quality to enter analyses while minimizing the risk of bias [29,69]. After the data passes QC, we will create a locked analysis pipeline for the creation of the TMS targets, thus removing any user bias or effect of experimenter expertise on target calculation. For all non-TMS target coordinate-related analyses, these pipelines will be used as shown or improved upon with the advent of better software and processing tools to ensure that the end analyses remain state of the art.

To take advantage of the outlined image standardization and large data set, the study will perform data harmonization with the aim of minimizing statistical bias from any sites or scanner vendors in addition to performing all analyses at the Nottingham site. Relevant work has been carried out to assess various approaches with differing MRI modalities via the Enhancing Neuroimaging Genetics through Meta-Analysis consortium [70]. ComBat [71] is one such tool of choice [72,73], as it has been shown to be effective in harmonizing diffusion [74], cortical thickness [75], and fMRI measures [76,77] while retaining relationships with known confounders such as age and sex. However, this field of research is highly active and, as such, the final decision of the best approach will be made before the start of the analysis to take advantage of the best and most robust tools available at the time of analysis.

Although this trial aims to investigate the effect of precision MRI-guided TMS treatment, this precision must not be carried out at the expense of treatment tolerability, and thus, participant comfort. For instance, when the location of the calculated treatment site brings about adverse events, such as facial nerve twitching and jaw clamping, we follow the protocol set out by Morriss et al [22]. This involves three different steps—(1) move the coil 1 cm (coil to be kept within 2 cm of the original site); if discomfort persists then (2) reduce threshold; and (3) revert to F3 spot, ensuring that the allocated treatment type is used. This final location is then used for stimulation throughout the course of treatment with the aim of improving the participant experience and maximizing adherence and tolerability.

It is hoped that the sharing of such a detailed MRI study protocol provides clarity to the methods used for the BRIGhTMIND trial and, as such, will aid and accelerate future studies and replications. In addition, this transparency will lend greater weight and credence to the results of the trial upon its completion. An important factor when the study has the potential to provide important insights into the mechanisms of TRD and the effect of TMS treatment.

## Appendix

Multimedia Appendix 1 Details of magnetic resonance scanning protocol.

Multimedia Appendix 2 Peer-review report from the Medical Research Council - National Institute for Health Research (MRC-NIHR) - (NHS, UK) (1).

Multimedia Appendix 3 Peer-review report from the Medical Research Council - National Institute for Health Research (MRC-NIHR) - (NHS, UK) (2).

Multimedia Appendix 4 Peer-review report from the Medical Research Council - National Institute for Health Research (MRC-NIHR) - (NHS, UK) (3).

## Abbreviations

ASL: arterial spin labeling
BET: brain extraction tool
BOLD: blood oxygenation level dependent
BRIGhTMIND: Brain Imaging Guided Transcranial Magnetic Stimulation In Depression
cgiTBS: connectivity-guided intermittent theta burst transcranial magnetic stimulation
CSF: cerebrospinal fluid
DICOM: digital imaging and communications in medicine
DLPFC: dorsolateral prefrontal cortex
DMPFC: dorsomedial prefrontal cortex
EPI: echo-planar imaging
FAST: FMRIB Automated Segmentation Tool
FC: functional connectivity
FNIRT: FMRIB’s nonlinear image registration tool
FSL: FMRIB Software library
GABA: γ-aminobutyric acid
GCA: Granger causality analysis
HDRS-17: 17-item Hamilton Depression Rating Scale
lCEN: left central executive network
LCModel: linear combination model
lMFG: left middle frontal gyrus
MEGA-PRESS: Meshcher-Garwood Point Resolved Spectroscopy
MRI: magnetic resonance imaging
MRS: magnetic resonance spectroscopy
NHS: National Health Services
NIHR: National Institute for Health Research
NLR: nasion-left-right
QC: quality control
rAI: right anterior insula
rsfMRI: resting-state functional magnetic resonance imaging
rTMS: repetitive transcranial magnetic stimulation
sgACC: subgenual anterior cingulate cortex
SPM12: Statistical Parametric Mapping v12
SUSAN: Smallest Univalue Segment Assimilating Nucleus
TBS: theta burst stimulation
TE: echo time
TMS: transcranial magnetic stimulation
TR: repetition time
TRD: treatment-resistant depression
WM: white matter
