# Supplementary material for: Connectivity-Guided Theta Burst Transcranial Magnetic Stimulation Versus Repetitive Transcranial Magnetic Stimulation for Treatment-Resistant Moderate to Severe Depression: Magnetic Resonance Imaging Protocol and SARS-CoV-2–Induced Changes for a Randomized Double-blind Controlled Trial
Source: JMIR Res Protoc. 2022 Jan 20;11(1):e31925. doi: 10.2196/31925 (PMC8814922; doi:10.2196/31925)
Supplement: Multimedia Appendix 1 [file resprot_v11i1e31925_app1.docx]

# Supplementary Materials

## Magnetic resonance scanning protocol

### T1-weighted volume

Nottingham GE Discovery 750

- Sagittal FSPGR Bravo (or equivalent)
- TR=8.156 ms, TE=3.172 ms, 1x1x1 mm
- FOV=256×256×156 or higher

Nottingham Philips Ingenia

- Sagittal T1 TFE
- TR=8.5 ms, TE=4.6 ms, 1x1x1 mm
- FOV=256x256x180

Newcastle Philips Achieva dStream

- Sagittal T1
- TR=8.3 ms, TE=4.6 ms
- FOV=256×256×180

London Siemens Prisma

- Sagittal FSPGR Bravo (or equivalent)
- TR=8.156 ms, TE=3.172 ms, 1x1x1 mm
- FOV=256×256×156 or higher

### Resting-state fMRI (with fixation cross, eyes open)

Nottingham GE Discovery 750

- Gradient echo EPI
- TR = 2s, TE = 32ms, flip angle=77°
- 35 slices, 3x3x3mm (with 0.5mm slice gap)
- FOV=192x192 mm
- Interleaved bottom/up
- Reverse encoding EPI scan immediately before scan
- 240 volumes

Nottingham Philips Ingenia

- Gradient echo EPI
- TR = 2.1 s, TE = 32 ms, flip angle=77°
- 35 slices, 3x3x3 mm (with 0.5mm slice gap)
- FOV=192x192 mm
- Interleaved bottom/up
- Reverse encoding EPI scan immediately before scan
- 240 volumes

Newcastle Philips Achieva dStream

- Gradient echo EPI
- TR = 2s, TE = 32ms, flip angle=77°
- 35 slices, 3x3x3mm (with 0.5mm slice gap)
- FOV=192x192 mm
- Interleaved F/H
- Reverse encoding EPI scan immediately before scan
- 240 volumes

London Siemens Prisma

- Gradient echo EPI
- TR = 2s, TE = 32ms, flip angle=77°
- 35 slices, 3x3x3mm (with 0.5mm slice gap)
- FOV=192x192 mm
- Interleaved bottom/up
- Reverse encoding EPI scan immediately before scan
- 240 volumes

### Cerebral blood flow maps using whole brain Arterial Spin Labelling

Nottingham GE Discovery 750

- pCASL 3D spiral acquisition
- TR=4632 ms, TE=10.5 ms, TI=2025 ms, flip angle=111°
- 36 axial slices, 1.875 x 1.875 x 4 mm
- FOV=240x240 mm
- eyes open

Nottingham Philips Ingenia

- pCASL acquisition
- TR=4800 ms, TE=15 ms, Label duration=1500 ms, post label delay=2000 ms, flip angle=90°
- 26 slices, 3.75 x 3.75 x 5.5 mm
- FOV=240x240 mm
- Eyes open

### MEGA-PRESS spectroscopy

Nottingham and Newcastle
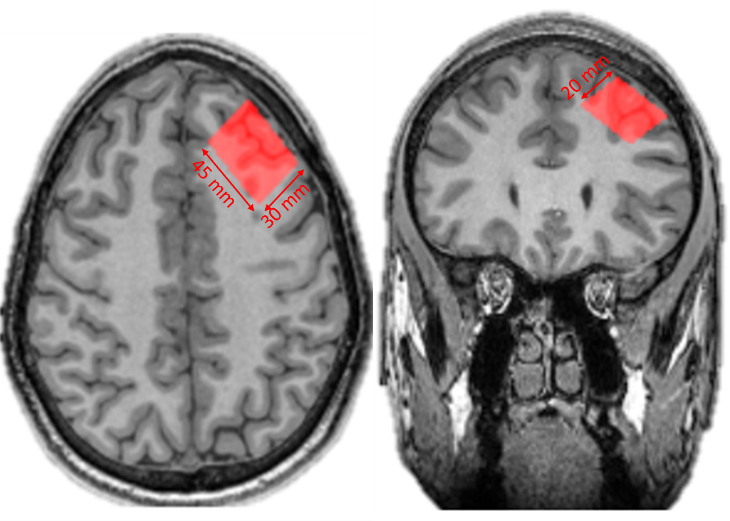


- DLPFC voxel placed in **LEFT** DLPFC
  - Dimensions should be:
    - 45 for A/P
    - 30 for L/R
    - 20 for I/S
  - Placement of voxel and shim box

should always avoid skull


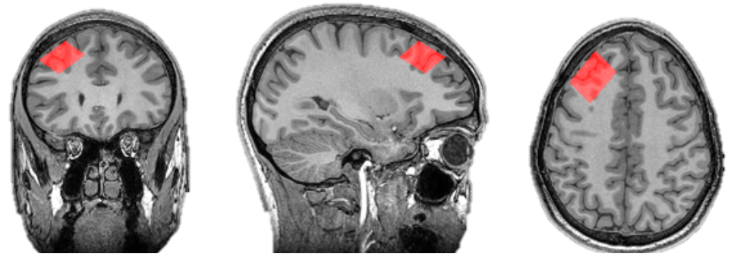


- TR=2000 ms, TE=68ms
- 320 averages
- Water suppression type and non-suppressed water spectra details:
  - Nottingham=CHESS (scan includes non-water-suppressed spectra, NSA=8)
  - Newcastle=CHESS (a separate non-water-suppressed spectra will be acquired, NSA=8)
    - Ensure voxel position, shimming and parameters are identical to water suppressed (except for NSA).
-
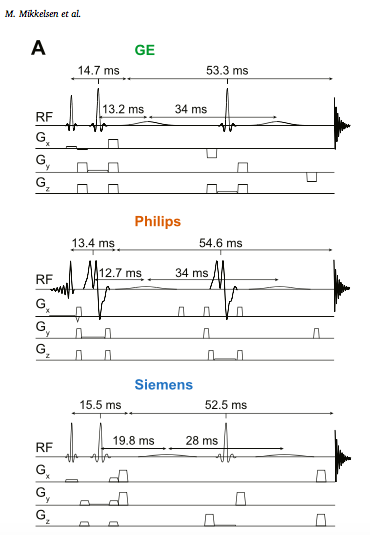
Scheme as described in Mikelsen *et al.*, Neuroimage 2017

### Diffusion-weighted imaging

Nottingham GE and Philips

- Resolution: 2x2x2 mm
- Field-of-view: 220x220 mm
- 64 directions (b=1000) with 5 b0 acquisitions
- 55 slices
- TR = 11 sec; TE = minimum
- flip angle =90
- phase = AP
- SENSE = 0
- Single b0 with reverse phase encoding with the same parameters with same TE as b1000

Newcastle Philips Achieva dStream

- Resolution: 2x2x2 mm
- Field-of-view: 220x220 mm
- 64 directions (b=1000) with 5 b0 acquisitions
- 55 slices
- TR = 11 sec; TE = minimum
- flip angle =90
- phase = AP
- SENSE = 0
- Single b0 with reverse phase encoding with the same parameters with same TE as the b1000

London Siemens Prisma

- Resolution: 2x2x2 mm
- Field-of-view: 220x220 mm
- 64 directions (b=1000) with 5 b0 acquisitions
- 55 slices
- TR = 11 sec; TE = minimum
- flip angle =90
- phase = AP
- SENSE = 0
- Single b0 with reverse phase encoding with the same parameters with same TE as b1000
