# Supplementary material for: Connectivity-Guided Theta Burst Transcranial Magnetic Stimulation Versus Repetitive Transcranial Magnetic Stimulation for Treatment-Resistant Moderate to Severe Depression: Magnetic Resonance Imaging Protocol and SARS-CoV-2–Induced Changes for a Randomized Double-blind Controlled Trial
Source: JMIR Res Protoc. 2022 Jan 20;11(1):e31925. doi: 10.2196/31925 (PMC8814922; doi:10.2196/31925)
Supplement: Multimedia Appendix 2 [file resprot_v11i1e31925_app2.pdf]

RESEARCH PROPOSAL EXTERNAL REVIEW OPEN COMMENTS

Review Questions

Question and Response
Importance, acceptability and potential impact of the proposed research?<br><br>The applicants have highlighted the importance of the economic and health burden of depressive illness to the wider community. Also, they have highlighted its often unsatisfactory treatment outcome, with various treatment modalities and the significant proportion of patients with treatment resistant depression. They could go further and discuss the potential cost offset for this intervention relative to other treatment technologies and existing stimulation technologies. This would help underline the potential "payback" from investment in funding this study.<br><br>If the intervention is effective the next movement in research in this area would be to define the most appropriate method and intensity of stimulation interventions in TRD. The applicants propose a comparative or "head-to-head" study against repeated TMS if efficacy is established. It must be borne in mind that existing drug treatments for TRD may have limited effectiveness, even in the best hands, and technologies such as ECT can prove unacceptable and may have limited duration of effectiveness. Stimulation technologies offer greater acceptability and lower risk than ECT, but again, duration of treatment response has been modest. An intervention that was proved to be effective and acceptable and had a longer maintenance of treatment gains would be a major treatment option for TRD. If it was proved to be superior to other technologies, this could lead to a new era in our treatment approaches.<br><br>The proposal is well-focussed on increasing our understanding of potential therapeutic mechanisms in depression. The study of cortical inhibitory function (and GABA/GLx)in a specific area after focussed low dose stimulation is an elegant method to assess these treatment effects and could be readily replicated. Allied to clinical correlates of treatment response this would help us understand neural network changes that underlie response and add to our understanding of depression. The applicants mention that it would aid their ability to predict response of depression by these technologies which would be a major therapeutic and research tool.<br><br>The only concern that I have in terms of acceptability is the intensity of the intervention. While fully accepting that this decision has had PPI involvement, I suspect that this was based on experience with the unblended, "active" treatment and it will remain to be seen how this is tolerated in a "sham" condition. Other than that aspect of treatment and assessment intensity, the applicants have gone to great lengths to discuss the acceptability and tolerability of stimulation.
Proposed research methods, recruitment and scientific quality?<br><br>This is a very well thought through and important proposal. Theta burst brain stimulation (TBS) may potentially have fewer side effects and a greater duration of effect than NICE recommended repeat TCMS (rTMS) used in treatment resistant depression (TRD). The study extends on knowledge and experience from an MRC funded pilot in Nottingham and is of great current importance in view of the costs and disease burden of major depressive disorder (MDD) and TRD are high. The MRC pilot suggest a longer treatment effect of TBS over three months than TCMS (rTMS).<br><br>The applicants also state that they intend to study patient qualitative views on rTMS or TBS. These have attracted little attention and will be assessed as a secondary aim and they will assess the

Question and Response
acceptability of treatment.<br><br>Comments:-<br>Background and rationale<br>I think it would be helpful to mention the other TBS pilot briefly, to spell out whether there were any differences in the effectiveness or drop-out or methodology to consider in their design.<br>The outline seems very good overall and I have mainly points for clarification and some suggested changes. There is a compelling and clear rationale of the burden of disease in TRD/MDD and if this adaptation of the new stimulation technology is superior and better tolerated, then the trial needs doing. The applicants could go further with the available NICE appraisal, recent evidence synthesis for rTMS and their pilot data and consider what the net cost advantage of the proposed TBS could be compared to other technologies. The applicants mention that TBS may be a better tolerated treatment than rTMS. What are comparative differences in expected adverse event/side effect burden of TBS and rTMS?<br>Methodology:-<br>The effectiveness of blinding in the sham TBS group is not fully discussed. My understanding is that this was challenging in the earlier rTMS studies of effectiveness. What experience have the applicants or others had in successfully adapting the sham TBS? I assume that this will have been developed from the experience of the rTMS trials, but as the applicants suggest, the differences in the treatment technologies as applied between rTMS and TBS may require assurance that this “sham” treatment is clearly defined and operationalised. The methods do not specify how the success of “blinding” will be assessed<br>The numbers to be recruited for the study seem testing but may be achievable and feasible and the applicants have addressed this potential issue to some extent in the outline. I assume that this study is of equal randomisation into each arm i.e. 80 from a total of 160, but this is not specified in the outline. With equal numbers per site this will require projected recruitment of 25-30 per each arm per each site over the study duration. Will the centres be unbalanced in terms of recruitment i.e. not equal numbers? This may aid recruitment and trial completion to the outline proposal targets.<br><br>I also wondered whether a very important study such as this should consider collaborating with other centres that could deliver the technology, to improve recruitment rates and assure against under-recruitment risks. This should be commented on by the applicants in a fuller proposal. It is recognised that this can have risks too, however, in terms of trial protocol adherence and differential recruitment rates between sites.<br><br>The diagnostic criteria used and TRD criteria are clear. The outcome measures for depression seem well justified and conventional and the functional and cognitive measures established. It would be helpful to also specify severity bands of the entrants e.g. moderate, moderately severe etc; in terms of HAM-D score parameters.<br><br>The outline proposal does not give a detailed account of trial governance. More details will be required of the trial steering group or board and its independence. Other ethical and issues it could help address are any unexpected drop-out rate, adverse event rate or obvious treatment superiority of treatment.
Research team, resources and research management?<br><br>The research team appears very well balanced in expertise and is multidisciplinary. They all are known for having strong research track records and the "trialist" members of the team have experience in MRC and NIHR funded randomised controlled studies, including their design, recruitment and successful delivery to completion. They are complemented by researchers with a strong basic science record in the technologies being used. The research team have a record of high

Question and Response
quality scientific paper output.<br><br>The addition of the Leicester Clinical Trial Unit is of great assurance in allowing the trial to be conducted to a high standard and is a strength. The proposed randomisation method is well thought through and appears robust. As I have already mentioned, in a fuller study proposal, it would be helpful to be much more specific about the methods of trial research and clinical governance. Specifically, the Trial Steering group, their independence and how trial protocol adherence is monitored/ It would also help address any potential ethical issues of the study e.g. obvious inferiority of a treatment, unexpected drop-out rates or adverse event rates.<br><br>The three centres appear to have adequate infrastructure and resources to deliver this trial and have established services for TRD and a significant research background. It is clear that the NHS and non-NHS trial resources have been carefully considered. As I have previously mentioned, it might be advantageous to identify one or two other collaborating centres to assure against risks of delayed recruitment.
Ethics and governance?<br><br>The applicants have gone to great pain to have PPI involvement in designing the protocol and the Plain English summary. However, potential trial subjects may need far clearer explanation of the placebo/sham arm and the possible expected adverse events (based on the previous rTMS research and pilot TBS RCTs)within the patient information leaflet (PIL).<br><br>My major ethical concerns are about the study burden, in terms of intensity of treatment and of assessments, especially for the sham group. Admittedly the sham group will still have their usual treatment plan and the experimental treatment is additional. I am unclear if this would be a deterrent to entry or increase the risk of drop-out, as the previous pilot did not have this arm. The applicants should clearly address these concerns in a fuller proposal and how this may need to lead to protocol adaptation. The supporting roles of the scientific officers from the CRN, research team and NHS team in helping to address and alleviate study burden need clearer description.<br><br>Other governance concerns revolve around the effectiveness of blinding and study group ascertainment in the "sham" group. If this proves to be high and early in an intensive study period could this affect drop-out? Other concerns are if there is a higher adverse event rate than expected? For these above reasons and also the issue of evident treatment inferiority in one arm, an independent Trial Steering Group should be in place (with relevant expertise in the technology used as well). It was not explicit that this was being considered in the proposal reviewed, but I am confident that the applicants will have already considered this action.<br><br>As well as addressing valid risks to the original trial protocol and inclusion/exclusion criteria and protocol adherence by GCP in each site, this could help address the above governance issues.
Patient and public involvement?<br><br>The applicants have obviously given a great deal of thought to this aspect of the proposal and a group has been established already and influenced the proposal. I was struck that the preference of this group was for a more intensive but briefer duration of intervention, which I found very interesting. It might be helpful to discuss what other treatment intervention models i.e. dosage and frequency which had been considered and why this decision was arrived at in the proposal. The applicants have considered patient acceptability as an important secondary aim.<br>The authors mention that there will be continued PPI involvement throughout the study.
Does the plain English summary give a clear explanation of the research?

Question and Response
I thought that this was very clear overall and would be easy to understand. I thought it was very well-written and do not have many comments. I felt that possibly some more explanation (brief) could be given of the term "placebo" and what it meant in this context of the study of the sham treatment arm. Possibly there could be clarification of the "paper and pen" tests used also.
Research outputs?<br><br>The publication track record of the authors is very strong and this would engender confidence about the potential proposed research outputs from the study. Technology and intellectual property considerations are discussed by the applicants. I think that the internal and external dissemination strategies are all relevant and the use of websites for dissemination. Possibly the applicants could be more specific on the main publications they anticipate and international conferences at which they envisage presenting. I also wondered if the article processing costs they mention would sufficiently support their output aims?
What are the key strengths of this proposal?<br><br>The area of treatment resistant depression (TRD) is of vital national interest from a scientific, clinical, health economic and social perspective. The research is therefore very timely. There are relatively few treatment options in this group and the emerging technology of magnetic stimulation shows promise (repeated TMS), but may only have short duration of efficacy. Similar issues may affect other interventions such as ECT. The refined technology used of theta burst stimulation (TBS) in the proposed study may promise a more sustained treatment effect and possibly a greater treatment effect than repeated TMS. If the proposed trial shows efficacy against a sham condition, the way would be open for a direct comparative study of TBS and repeated TMS. The study group and diagnostic and clinical entry criteria are well defined. The outcome measures used are well established and will be helpful in later evidence synthesis in this area.<br><br>This is a large study and will be influential and one of the larger studies in the literature. The numbers included in the study are substantial and will give clarity over the effectiveness and tolerability of this intervention and particularly, longer term effectiveness. The study duration is a strength in this proposal. The study is not that expensive in my view, equating to around £8000 per entrant, considering these are a difficult to treat and treatment resistant group. This shows considerable potential for payback of the investment in clinical, scientific and economic terms, if it is effective. The costs of ineffective treatment for this group needs to be considered in this context.<br><br>The study also is well focussed on the proposed underlying mechanism of therapeutic action (GABA and inhibitory cortical function) with magnetic spectroscopy methodology to assess these changes as clinical correlates of response status and assessment of other correlates e.g. cognitive correlates. This may help increase our understanding of neural mechanisms in depression and response predictors.
What are the key weaknesses of this proposal?<br><br>These are addressed within the comments on previous review questions. In summary, these are:-<br><br>1.Are there sufficient sites - whether three sites is sufficient, bearing in mind that a significant number of the existing TRD caseload at the sites will need to be successfully recruited to meet the study requirements. Either the study duration will need to be considered to extend the recruitment arm or other sites may need to be involved to complete to the envisaged study deadline. or whether consideration to the addition of one or two extra sites with comparable resources and expertise.

Question and Response
2. Concomitant medication - the applicants should be clear whether potentially GABA modulating drugs are excluded.<br><br>3. Blindness - there should be assurance regarding the design and operationalisation of the sham treatment. In earlier TMS studies the blind condition was possibly problematic. An assessment of the robustness of the blindness condition is needed in this study.<br><br>4. Trial Steering Group - it is recognised that the Leicester Clinical Trials Unit has an active role in the trial governance but an independent reference or steering group would help address ethical and other governance issues of the trial. it may be that this has not been emphasised in the proposal due to constraints of space, but these mechanisms should be clarified and external collaborators willing to be involved should be identified.
Do you have any questions for the applicants that you would like the opportunity for the applicants to respond to prior to the proposal being considered by the funding board?<br><br>No, these are addressed within my comments in the review questions.

Applicant Response To External Review

Applicant Response

Overall response to reviewer of EME TBS versus sham TBS TRD bid.

Thank you for the reviewer's comments. The reviewer pointed out the huge public importance of treatment resistant depression, how stimulation technology such as this with a longer maintenance of effect "could lead to a new era in our treatment approaches", and the proposal was "well thought through and important", "inexpensive given the high cost of ineffective treatment" and has a well-focussed proposal to study the underlying mechanism of action. We wish to address the following points that were raised about the proposal by the referee.

1. Cost offset of intervention.

We agree with the reviewer that we may have undersold both the importance of treatment resistant depression as a health problem and the potential benefit of TBS in cost savings for the NHS. In terms of years lived with disability, major depression is the 2nd biggest health problem in the world; and it is the 3rd biggest in relation to disability adjusted lived years once effects of depression on cardiovascular mortality are considered. Major depressive disorder is an expensive condition with direct costs of £1·7 billion per year (through use of services, mainly non-psychiatric National Health Service [NHS] use, third sector and social service delivery) and indirect costs of £7·5 billion per year (mainly through lost employment) in England alone in 2008 (McCrone et al, 2008). These costs escalate substantially as 7% of patients with depression in secondary mental health care develop a chronic unremitting course, for whom evidence indicates will have particularly high rates of admissions to hospital, functional impairment, and suicide. Serious depression can be lethal once people develop other serious medical illness and a recent RCT of patients with persistent depression found that two thirds of such patients had a serious medical illness. Treatment resistant depression (TRD) affects 1% of the general population and therefore it is as prevalent as other serious mental illness such as schizophrenia and bipolar disorder. Previous research by the authors show that such patients with TRD need to be seen in primary care on average at least once per month and cost £4,000 per year to manage often for many years (median of seven years in one recent multicentre RCT). Much of this care is aimed at preventing deterioration in function rather than improving clinical outcome.

To keep people with TRD well they are given treatments that are unpopular with the potential for serious permanent side-effects e.g. electroconvulsive treatment with the risk of permanent memory deficit, atypical antipsychotic drugs precipitating diabetes, antidepressants raising the risk of stroke through intracranial haemorrhage. TBS has a minimal side-effect burden and is not known to cause any serious adverse effects.

rTMS has a minimal duration of clinical effectiveness against depression symptoms lasting for one month in about 38% of people with TRD offered it but the benefits are greatly diminished by 3 months. Yet rTMS may be a cost effective treatment of TRD compared to antidepressant treatment, the current standard NHS treatment for TRD (Nguyen KH and Gordon K, 2015). Our pilot and some other independent trial data on TBS suggests that compared to rTMS, TBS has a longer duration of clinical effect of at least 3 months and showed a clinical benefit in twice as many participants than those offered rTMS with fewer side-effects. Such effectiveness would substantially reduce the need for ongoing health and social care, reduce death from not only suicide but also death and serious disability from comorbid physical illness, and reduce the need to utilise other treatments with a high

**Applicant Response**
potential for iatrogenic harm. Given that TBS is safe and can be given 2 or 3 times per year, a conservative estimate of savings for this common mental health problem may be in the order of £1,500 per patient per year in at least 50% of people with TRD offered the treatment (please note than in our pilot 88% responded at 3 months), plus additional knock on reductions in costs in relation to the family that may accumulate substantially over time, bearing in mind that TRD is principally a problem of working age and economically active adults with families.<br><br>2. Recruitment to a study with a sham intervention.<br><br>We will offer all participants at the end of the study an expert review of their treatment if they wish. The TBS or sham TBS interventions we offer is in addition to all routinely available treatment, and except for a small number of medications that are infrequently prescribed, we will not deprive anyone of any routine treatment. One option would be to offer everyone who has not improved with either TBS or sham TBS at 6 months, a course of TBS but we do not propose to do this because the treatment does not have proven efficacy (which we hope to show in this RCT). We will address this issue again in a full application where we will consult more widely with our PPI group whether the option of offering rTMS at 6 months to all those who have not improved and wish to try it, would be an incentive to recruitment. rTMS is a NICE supported intervention to all those offered sham TBS. The additional cost will be approximately £63K.<br><br>3. More detail on TBS pilot.<br><br>There were no additional methodological issues than are raised by the reviewer from the pilot study. The recruitment rate of all participants approached to take part in the pilot study was 59%. There were twice as many side-effects in the rTMS arm than the TBS arm and these were mild self-limiting headache of brief duration in a small proportion of individuals on some occasions. There was only 10% drop-out from treatment, 10% drop out from follow up and 10% did not take part in a second MRS scan at 3 months. There was no differential drop-out or loss to follow up by treatment arm although the numbers of participants were small.<br><br>4. Unblinding in sham TBS<br><br>Since TBS utilises a lower frequency of stimulation, the prevalence of side-effects such as headache and scalp discomfort are usually of the order of 4% and 2% respectively as opposed to 3% and 2% respectively with sham TMS (NICE review). Therefore unblinding because of differential rates of side effects in a TBS versus sham TBS RCT should not be as problematic as in high frequency rTMS versus sham TMS RCTs where the prevalence of headache and skin discomfort side-effects with rTMS can be as high as 10%.<br><br>A recent review of sham TMS has highlighted a number of possible approaches to delivering sham TMS. The best approach in terms of blinding is to use a purpose built sham TMS coil in combination with electrical stimulation of the skin since no attenuation of the magnetic field is possible but participants will experience all the sensory experience of TMS or TBS (Duecker and Sack, 2015). We will adopt such an approach.<br><br>Furthermore if we can assure participants that there is likely to be no differences in side-effects, which are in any case temporary and very mild, then participants are less likely to draw conclusions about which treatment arm they are in on the basis of any sensory experiences or changes in their body they may have. Such an approach should reduce the risk of drop-out and unblinding.<br><br>Only the trial co-ordinator and nurse administering the TBS or sham TBS treatment will know the treatment allocation so that the participants, NHS staff and raters of outcome are all blinded.

Applicant Response
We will ask both each participant and each rater to guess treatment allocation on a five point scale (definitely sham, possible sham, unsure, possibly TBS, definitely TBS) on a weekly basis during TBS and at each follow up assessment. Any cases of definitely being sure of their allocation will be explored qualitatively at interview to check the basis for such an opinion, whether or not it is correct. If unblindings occur, we will retain the participant in the intention to treat analysis by asking a second blinded rater (a CRN clinical scientific officer) to carry out all further follow up.<br><br>5. Equal randomisation and balancing of recruitment across sites.<br><br>As stated on page 7 of the application, participants will be equally allocated. However, if shortlisted for a full application, we will consult with our statistics experts and trial methodologists as well as PPI representatives and NHS clinicians who might refer into the study about a strategy that randomises participants in a ratio of 3:2 TBS to sham TBS at all sites. The sample size would only be very modestly increased from 144 participants to 155 participants, and would be worth doing if consultation with PPI and clinicians gives us feedback that recruitment rates are likely to be increased as a result. Psychologically we think participants to the study and their clinicians are more likely to consent and remain in the RCT because more likely than not a participant will be randomised to the active treatment. Such an approach would lead to only slightly unequal size groups that should not compromise blinding and there will be a substantial sample in each treatment arm to estimate the treatment effect. Unequal randomisation with a greater number of TBS participants will also enable a more detailed examination of the side-effects (which are relatively rare) and acceptability of the TBS intervention, and also moderators of response within the TBS group, a step towards predicting who might benefit most from TBS. Such an approach is sometimes used in drug versus placebo randomised controlled trials. We can review this randomisation approach also in the internal pilot, even considering 2:1 randomisation, if qualitative interviews and feedback suggest that such an approach would randomise a greater proportion of eligible people with TRD.<br><br>On p 7 of the application we have already outlined a proposal that will recruit differentially across centres because of size and available resources at each site. On page 7 of the application, we have already outlined that we will also stratify our randomisation by centre, and the analysis will adjust for centre effects as stated on page 8 of the application.<br><br>6. Severity of TRD.<br><br>We will categorise each participant according to Zimmerman et al (2013) criteria for the severity of depression. Each participant will be at least moderate severity of depression at baseline and will be categorised into moderate, moderately severe and severe. The analysis plan on page 8 already includes baseline severity of depression measured on the primary outcome measure (HDRS-17) as a continuous scoreas well as treatment centre to estimate the treatment effect on the primary clinical outcome measure. The purpose of the categorisation by severity is to help communicate the results of the study to clinicians who are more familiar with these categories than the raw HDRS score. We will not randomise by severity of depression as stratification and minimisation on too many characteristics can lead to greater imbalance between the treatment arms on characteristics that may either affect outcome or the understanding of the underlying mechanism of effect.<br><br>7. Trial governance.<br><br>Given space restrictions we did not provide details of the independent trial steering committee and data monitoring and ethics committee that we will appoint. We will appoint a Trial Steering Committee (TSC) chaired by an external independent psychiatrist with experience or running multicentre trials and TMS studies, along with an external neuroimaging expert, a statistician, and an independent PPI representative. The meetings will be held with the attendance of the research team excluding the chief investigator, a centre lead and PPI representative and the study statistician. However, the meeting

**Applicant Response**
will also exclude the study team during key deliberation and decision-making. It will report to the funders and sponsors of the study directly as well as the chief investigator. There will also be an independent data and monitoring ethics committee chaired by a psychiatrist with trials expertise in depression, another independent statistician and another PPI representative. The chief investigator, study statistician, PPI lead and trial co-ordinator will also attend part of these meetings but again will also meet independently of the research team. The DMEC will report to the TSC who will make any final decisions independent of the research team. We will not approach any potential members of the TSC or DMEC unless we are shortlisted for a full application. In addition we will register our trial before the first participant is recruited, publish our protocol once the internal pilot is completed, and publish our statistical analysis plan before the analysis is started.<br><br>8. Supporting roles of research team, NHS team and CRN scientific officers.<br><br>The Clinical Scientific Officers of the Clinical Research Network, PPI network, NHS staff working on the project and research team will publicise recruitment to the study in the secondary care mental health services, primary care, third sector groups and also through service user organisations. Often we find that directly advertising such studies to service users and primary care can lead to higher rates of recruitment even if participants with TRD are in secondary care, because people with TRD are seen more frequently in primary care and the third sector (where the burden of care may often lie) than in secondary care. Research assessments will usually be done by research staff but we will designate one to two CSOs from the CRN to work on the project at each site to complete assessments whenever necessary due to the unavailability of research staff or for technical reasons such as rare cases of unblinding, or personal preference of a participant to see a RA of a particular background. The NHS staff will play no role in the research assessments but will help recruit participants and deliver both TMS treatment and other concomitant treatment. As outlined in the proposal SL, a NHS psychiatrist with considerable clinical and research expertise in TBS and TMS, will spend some of their time at each centre along with the appointed trial co-ordinator to ensure consistency of approach in terms of recruitment, treatment and research assessment at all centres. All staff involved in delivering the study will have up to date Good Clinical Practice Training.<br><br>9. PPI involvement.<br><br>The reviewer asked what options PPI were given concerning the delivery of TBS and sham TBS. PPI preferred a more intensive course of TBS 4 or 5 times per week over 4 weeks than 3 times per week over 6 weeks as we originally proposed. We also discussed approaches to sham treatment and explanations of it. They reviewed our previous and proposed patient information sheets, and recruitment and consent procedures; they thought that we correctly represented the research and they fully supported our expalantion that people receiving TBS or sham TBS sometimes improved but sometimes did not. They thought that people with TRD would accept this if there was such equipoise, and would welcome the offer to meet with clinical experts in TRD. They preferred to have the option of carers coming with them for treatment and scanning so we have budgeted for this. In addition to academic presentations we will work with PPI to share our results once they are peer reviewed and published with user groups and national mental health charities e.g. Depression Alliance through face to face, video clip and printed forms of dissemination as the PPI group requested.<br><br>10. Plain English summary.<br><br>If we are invited to make a further application we will describe the placebo and give more detail on the paper and pen tests as suggested by the reviewer.<br><br>11. Publications.<br><br>In terms of the main trial outcomes we will aim to publish in a main general journal that has open

Applicant Response
access e.g. Lancet, BMJ or a top specialist psychiatry journal e.g. Lancet Psychiatry, Am J Psychiatry. In terms of the mechanism of action we will publish in a top neuroimaging journal e.g. Neuroimage or biological psychiatry journal e.g. Biological psychiatry. We will publish the results of the acceptability research either with the main trial report or separately in an open access journal that publishes qualitative research methods when relevant to clinical practice e.g. Journal of Affective Disorders, BMC Psychiatry.<br><br>12. Number of sites.<br><br>We will not recruit additional sites because to do so would prohibitively increase costs. With the company that produces the TBS equipment and with the agreement of senior clinical staff, we will if necessary recruit in a second neighbouring trust to each site using no additional research staff but additional clinical support officer time. Potential second sites would be close enough to add modest additional costs for scanning (additional travel and subsistence) while participants could elect to receive treatment at that second site or the main research site. Therefore, potential additional sites close to Nottingham that are willing to provide TBS are Leicestershire Partnership Trust, close to Northampton mental health services in Milton Keynes where there is already a satellite TMS clinic delivered by Northampton mental health services, and Tees Esk and Wear NHS Trust close to Newcastle.<br><br>In the original pilot RCT, recruitment of all participants was done through one consultant with no research assistant or clinical scientific officer support. We have assumed only a similar rate of recruitment to the pilot RCT. We expect recruitment to be achieved at a similar rate to that we have achieved before in Nottingham and Newcastle in randomised controlled trials of active treatments for TRD or chronic depression involving placebo or no active treatment other than treatment as usual. These studies benefited from research assistant and clinical support officers from the Clinical Research Network that were not available in our pilot RCT but would be available in the proposed RCT. At Nottingham and Northampton we aim to recruit 15% of our existing TRD service users who have expressed an interest in TMS as a treatment per year. The Newcastle site does not have a NHS TMS service but does have a TRD service with many service users willing to try TMS. We do not think the proportion of people with TRD willing to participate in our RCT would be any lower in Newcastle than at the other sites.<br><br>We have considered lengthening our recruitment period but such an approach would add considerably to the cost of the study so we have decided not to propose this.<br><br>While it is true that sham TMS studies may be more difficult to recruit to than RCTs of two active treatments we think we can mitigate the risk of low recruitment by:<br><br>1. As suggested by our PPI group explaining that both treatments have been associated with improvement as well as non-improvement in outcome, and we do not know how effective each treatment is so we are conducting this study. Both treatments are associated with very few side-effects and have an excellent safety record. While they are taking part they continue to receive medication and other treatment e.g. social care as they currently do. They will also receive expect assessments in TRD through the study and at the end of it. They will know at the end of the study what treatment they received and will have a chance to discuss with a clinical expert as well as their own team what the next steps for treatment will be. We will consider offering everyone who wishes to receive it and has not responded to TBS or sham TBS, a course of rTMS after the final 6 months outcome assessment.<br><br>2. We will monitor recruitment and conduct qualitative interviews to understand any issues related to non-participation, randomisation or drop-out and then attempt mitigate the risk with PPI, clinician and methodological support. All decisions will be checked with independent TSC and DMEC

Applicant Response
who will include experts in all aspects of the trial.<br><br>3. We will use approaches that are unlikely to compromise blinding or increase drop-out (thereby leading to the recruitment of an increased sample size) through unequal or different side-effects with different treatments. We will also consider unequal randomisation as a method of boosting rates of recruitment if equal randomisation to active treatment or sham treatment proves unpopular with service users or their referring clinicians in this distressing condition.<br><br>4. We will call upon additional CSO support and if necessary recruit from a second mental health service close to each of the three sites, approximately doubling the population from which we can recruit our sample.<br><br>13. Concomitant medication.<br><br>The research literature on the effects of medication on GABA/glutamate in MRS scanning is not clear cut. The best evidence suggests that gabapentin may have an effect on the estimation of GABA but not glutamate (Cai et al, 2012) so there is the potential for drugs that modulate GABA directly or indirectly to affect the estimate of GABA. Therefore we will exclude anyone using rarely prescribed potentially GABA modulating drugs such as lamotrigine and gabapentin. Other drugs that could potentially modulate GABA and are frequently used in TRD will be kept at a stable dose for 4 weeks before each GABA/glutamate MRS scan, namely SSRI antidepressants (indirectly a relationship has been suggested), pregabalin and hypnotic drugs. We cannot exclude participants who are prescribed this medication because the randomised controlled trial would not be feasible and would have very little external generalisability to clinical practice. There is no evidence that stable doses of these drugs have any effect on GABA/ glutamate MRS scans, and in any case we require stable doses of these drugs during the randomised controlled trial to accurately estimate the clinical effects of TBS versus sham TBS on the primary clinical outcome. Therefore we are not adding any additional burden to the participants through our examination of the mechanism of action of TBS. No effects of stable medication were observed in our pilot study, and if present at all, they are relatively small compared to the effects from the severity of depression at baseline and treatment effect from TBS on estimates of GABA in MRS scans.<br><br>References (only included if they are not cited in our first proposal reference list)<br><br>Cai K, Nanga RPR, Lamprou L et al. The impact of gabapentin administration on brain GABA and glutamate concentrations: a 7T ^{1}H-MRS study. Neuropsychopharmacology 2012; 37: 2764–2771.<br><br>Duecker F, Sack AT. Rethinking the role of sham TMS. Frontiers in Psychology 2015; 6: 1-5.<br><br>Nguyen KH, Gordon LG. Cost-Effectiveness of Repetitive Transcranial Magnetic Stimulation versus Antidepressant Therapy for Treatment-Resistant Depression. Value Health. 2015;18:597-604.<br><br>Zimmerman M, Martinez JH, Young D, Chelminski I, Dalrymple K. Severity classification on the Hamilton Depression Rating Scale. J Affect Disord 2013; 150: 384–88.
