# Supplementary material for: Connectivity-Guided Theta Burst Transcranial Magnetic Stimulation Versus Repetitive Transcranial Magnetic Stimulation for Treatment-Resistant Moderate to Severe Depression: Magnetic Resonance Imaging Protocol and SARS-CoV-2–Induced Changes for a Randomized Double-blind Controlled Trial
Source: JMIR Res Protoc. 2022 Jan 20;11(1):e31925. doi: 10.2196/31925 (PMC8814922; doi:10.2196/31925)
Supplement: Multimedia Appendix 3 [file resprot_v11i1e31925_app3.pdf]

RESEARCH PROPOSAL EXTERNAL REVIEW OPEN COMMENTS

Review Questions

Question and Response
Issues for consideration by the board<br><br>This team have considered previous feedback from the board and redesigned parts of their study to take into account those issues raised. As a result the proposal is strong and well considered. It is underpinned by an experienced team who have piloted, and assessed the feasibility of both the randomised trial and the mechanistic sub-study thoroughly.
What are the key strengths of this proposal?<br><br>Excellent team with a strong background in the technology.<br>An infrastructure well equip for delivery of both the treatment and the research.<br>Good pilot and feasibility work in preparation.<br>Good links to the CTU.<br>Well worked power calculations and appropriate analysis plan.<br>Well powered and competently designed main RCT (I do not have sufficient knowledge of the mechanistic study to pass comment).<br>The inclusion of a short piece of qualitative work on user experience to help evaluate the utility of either arm in the case where both treatments were judged to be equally effective was a welcome addition.<br>The research costs are reasonable (total per participant costs of less than £5000 pp, with the mechanistic study yielding interesting information from both arms).
What are the key weaknesses of this proposal?<br><br>The health economic element is not articulated well and feels like a tack-on, the analysis detail in the main protocol seemed a little too generic. This HE evaluation could be a very valuable source of information on which to base commissioning decisions so it would seem a shame to underplay its potential. In a similar vein, it would have been good to see mention of a small process evaluation around implementation.<br>There were no design or statistical weaknesses that I could detect although the treatment of missing data values could always do with more careful consideration in the final statistical analysis plan.
Does the plain English summary give a clear explanation of the research?<br><br>I understood the research from the plain English summary, but the average character count was 5 per word and the average sentence was 20+ words. It would be possible to improve its readability and comprehensibility prior to publication by a good sub-editor.<br>The contents gave a good explanation of the background and the RCT.
Do you have any questions for the applicants that you would like the opportunity for the applicants to respond to prior to the proposal being considered by the funding board?<br><br>I have no further questions for the applicants as their application was comprehensive and clearly written.

MRC Medical Research Council

NHS National Institute for Health Research

RESEARCH PROPOSAL EXTERNAL REVIEW OPEN COMMENTS

Review Questions

Question and Response
Issues for consideration by the board<br><br>I feel that if the minor clarifications mentioned (under weaknesses) are answered that the trial should be funded and supported. I think that a letter of clarification would be sufficient. I also feel that a letter from Birmingham stating the support and expertise that they can give to the trial would be helpful.
What are the key strengths of this proposal?<br><br>This is now a much stronger proposal and a lot of preparatory work and thought has been given to the study. The study will help determine whether or not there is an advantage over an existing recommended treatment by NICE, There appears to be a very clear selection process and the research group have established TRD referral networks and prior trial experience in this area. A pilot study has already been performed and this establishes feasibility of the study. The team now have four centres, with the option of a fifth, so risks to recruitment are reduced.<br><br>There is a clear methodology for TRD assessment, measures and study criteria. There are clear exclusion criteria, it is appropriate that this now includes GABA modulating medication.<br>DSM comorbidity has been considered in exclusion, but (as mentioned in weaknesses)a plan of secondary analyses of any "allowed" comorbidties should be considered. The study has very clear outcome measures and study treatment outcomes and clinical correlation with hypothesised network changes. The team will use well respected outcome measures that can be directly compared with other data sets and discussion of inter-site training has been mentioned. Standardised self-rated measures are used for other outcomes.<br><br>Recruitment from secondary and primary care is pragmatic and used for feasibility reasons, I presume. Still it would be helpful to clarify whether any cohort differences are expected, or outcome differences. How many for example have had previous secondary care contact but have disengaged and are now managed in primary care?<br><br>Use of the Leicester site for trail co-ordination, established CRN links and preparatory work for the trial. Trial governance seems well thought out - but after the recruitment phase and at later points of the study, what would lead to trial stopping or redesign of protocol or SOPS? There is very well evidenced and advanced public and patient involvement and experience of this from previous trials.<br><br>Costs are very well justified and this is in real terms a relatively inexpensive trial and represents good value for investment.<br><br>The team is very well-balanced and have the required wide range of skills and experience. They have a history of successful collaboration and recruitment and of high quality published outputs.
What are the key weaknesses of this proposal?<br><br>This is a greatly strengthened proposal. Rather than definite weaknesses there are a few issues that need greater clarification. In the revised protocol there is no sham group, as discussed by the applicants for ethical reasons. It would be helpful to have a more detailed justification of the scientific

Question and Response
and ethical reasoning behind this change in protocol with a further definitive statement why this is not necessary i.e. pilots or RCTs already published and extent of evidence base.<br><br>In recruitment, analysis of any possible selection biases between sites and inter-site differences in protocol adherence could be addressed by the Trial Steering Group, possibly the applicants could give greater assurance om whether this is a possibility or unlikely.<br>For the entry criteria, I take it that moderate severity is determined by the standard HAM-D cut-off criteria e.g. 15, 17,21 etc., but clarification is important, Will the randomisation process and analysis look at severity strata.<br><br>Study burden is still an issue for recruitment and retention, can any flexibility be brought in to the protocol and SOPS to aid retention or is it not possible?<br><br>Analysis of extraneous treatments e.g CBT, etc and of other possible comorbidity not exluded e.g. trauma, personality disorder should be discussed.
Does the plain English summary give a clear explanation of the research?<br><br>This is an excellent summary of a complex study. Great care has been taken to explain randomisation, what exactly will happen and potential benefits and risks of the study. The information should be readily accessible for lay groups and potential study entrants.<br>Use of Leicester site and CRN for trial<br>DSM comorbidity considered (but see above)<br>Standardised self-rated measures<br>Very good to have contingency of extra site in Birmingham<br>Keeping most medication (except GABA modulating) and<br>Team well-balanced.
Do you have any questions for the applicants that you would like the opportunity for the applicants to respond to prior to the proposal being considered by the funding board?<br><br>I only have one, which is if the Birmingham or London centres cannot be available for any reason at the proposed start of the trial, are there any other contingency plans for other centres?

MRC Medical Research Council

NHS National Institute for Health Research

RESEARCH PROPOSAL EXTERNAL REVIEW OPEN COMMENTS

Review Questions

Question and Response
Issues for consideration by the board<br><br>This is a potentially interesting study seeking to establish whether a novel form of rTMS, featuring MRI guidance and a briefer 'theta-burst' stimulation protocol, performs better than the standard US FDA-approved protocol in treating medication-resistant depression. The study has a commendably large recruitment target of 368 patients, which may (just) be feasible across 4 sites in the time allotted. Patient inclusion and exclusion criteria and outcome measures are reasonable and match standard practices for such a study fairly well. The mechanistic side of this proposal is quite strong and features interesting studies of how rTMS affects the network activity of the brain on functional MRI, as well as the neurochemistry of the brain on MRS. That said, there are a couple of features of the design (particularly the novel versus comparator intervention) which may contain critical flaws that would preclude meaningful interpretation of the findings.<br><br>Major issues:<br><br>- As noted in the 'Weaknesses' section, the critical design flaw in this study is that the cgiTBS intervention differs in two potentially important ways from the standard intervention: different protocol (theta-burst), and different stimulation site (fMRI-guided). As such, there is no way to disambiguate whether a superior effect for cgiTBS stems from higher potency of theta-burst stimulation or from more precise, individualized targeting of the stimulation site. The real-world impact of this is that one might falsely conclude that every patient presenting for rTMS will need to undergo fMRI before treatment in order to maximize the chances of success. If so, rTMS may have trouble making a meaningful reduction in the 2% of the population with treatment-resistant depression. The present study design therefore runs the risk of making it less clear, rather than more clear, whether rTMS is a value proposition for the public health system as a whole.<br><br>- A second major technical issue is that the MRI-guided stimulation site may, in some patients, be almost the same as the non-MRI-guided stimulation site; in other patients, the MRI-guided site could potentially be difficult to identify at all (as detailed below). Thus, it may be difficult to randomize patients in a meaningful way to MRI-guided versus non-MRI-guided stimulation arms.<br><br>Minor Issues:<br>- The authors describe pilot data for 'iTBS', which is normally considered an excitatory protocol and refers to a pattern of 2 s on, 8 s off x 20 trains of 50 Hz triplet pulses. However, their intervention protocol described 5 runs of a 40 s continuous train of 50 Hz triplet pulses - this would normally be considered 5 runs of continuous TBS or 'cTBS', generally considered an inhibitory protocol. cTBS, in published work and in our own experience, has not performed as well as iTBS. It would therefore be helpful to clarify if the authors' promising pilot work is from the same 5x cTBS protocol they are proposing to use here, or from 'true' iTBS as described above, as these two protocols are reckoned to have opposite effects. It would also be helpful to clarify why they appear to refer to their protocol as 'iTBS' when its parameters appear closer to cTBS repeated 5 times.
What are the key strengths of this proposal?<br><br>There are several encouraging strengths to this study:

Question and Response

- Theta-burst rTMS has the potential to dramatically improve the real-world utility of rTMS as a tool for making meaningful reductions in the 2% of the population with medication-resistant depression, at reasonable cost. As such, the potential impact could be high

- There are so far very few large-N studies to establish the relative efficacy of theta-burst stimulation over conventional longer protocols (although at least 1 large Canadian study of conventional 10 Hz vs short theta-burst rTMS is near completion, and will likely be published several years before this study is complete).

- The use of neuroimaging for individualized guidance is a 'gold-standard' approach for precision (although if the treatment truly proves to require MRI in every patient, its real-world impact may be reduced significantly)

- The enrolment of 368 patients would position the study as one of the largest brain-stimulation studies yet conducted (although a recruitment period of just 25 months is rather ambitious for such a sample in a 4-site study and would require sustained recruitment rates of ~1 patient per week per site - not unheard of, but worth providing supportive records to demonstrate feasibility)

- The team appears to have good experience in brain stimulation, depression, and neuroimaging

- Patient recruitment plans, inclusion and exclusion criteria, and outcome measures appear reasonable (although the intervention and comparator aspects of the design may have a critical flaw, as below)

- The collected data (functional MRI and MRS) in such a large patient sample would be a rich source of insight into the mechanisms of rTMS at the neurophysiological level

- The authors' hypotheses regarding DLPFC-DMPFC connectivity, DLPFC-insula connectivity, and prefrontal GABA (on MRS spectra) as mechanisms of action are intriguing and backed by interesting pilot data

- Economic and quality-of-life analyses are an important adjunct to the efficacy and mechanistic objectives of the proposal

What are the key weaknesses of this proposal?

There are some problematic features with both the strategy and the design of the study as presently proposed:

- The most critical design issue is that the novel intervention (cgiTBS) differs from the standard comparator intervention (10 Hz rTMS) in more than one way that is hypothesized to affect efficacy. Not only is the stimulation protocol different (theta-burst rather than standard 10 Hz), but the stimulation site is also different (fixed F5 location versus functional MRI-guided location). In the event that the study shows superiority for cgiTBS over conventional stimulation, there is no straightforward way to determine whether the improved outcomes arise from the theta-burst pattern of stimulation, or the more individualized targeting of the stimulation site, or some combination of the two.

- This issue has the potential to diminish the real-world impact of the study: for example, if the benefits are actually from the theta-burst stimulation and not from the fMRI-guided fine-tuning of the

Question and Response
target, one might falsely conclude that all patients need to undergo a costly and limited-access fMRI scan in order for rTMS to work properly.<br><br>- The requirement for a functional MRI session in every patient would markedly reduce the practicality of rTMS as a strategy for making meaningful reductions in the 2% of the population with treatment-resistant depression, so we would want very clear evidence on exactly how much benefit is conferred by fine-tuning the target using rTMS; the present study design does not enable us to determine whether any added benefits are from the fMRI guidance versus simply the theta-burst pattern of stimulation.<br><br>- There are also two practical problems with randomizing patients to fMRI-guided targeting versus a standard location:<br><br>First, for a substantial proportion of patients, in our experience, the fMRI-guided target ends up being less than 5mm from the fixed target in any case, so randomizing them to fMRI guidance ends up giving them more or less the same site as they would have had if they had been randomized to the fixed target (since the inter-session and inter-operator errors are usually on the order of 4-8 mm in any case). I do not see an easy remedy for this issue aside from enrolling a very large number of patients, treating them all according to some standard scalp heuristic generating high variance of sites across individuals, and then comparing outcomes as a function of the rTMS coil's distance from the fMRI-guided 'ideal' target in each person, having controlled for the many other variables that might also affect outcome. Logistically this would be rather tricky.<br><br>Second, for a subset of patients (up to 30% in our experience), connectivity patterns to the DLPFC have the opposite sign to the typical patient (e.g., positive connectivity rather than negative connectivity), or the nearest site in the frontal lobe with the desired connectivity sign ends up being well outside the DLPFC. It is not clear how the applicants propose to handle cases of patients who have the 'wrong' sign of connectivity in the target region, or whose connectivity target lies outside the DLPFC as typically defined.<br><br>In summary, although the successful use of neuroimaging for individualized rTMS targeting is a 'holy grail' for the field, and although theta-burst stimulation is a very promising innovation among rTMS protocols, the incorporation of both of these features simultaneously into the randomization is a critical conceptual flaw in the design. Moreover, if neuroimaging actually does turn out to be required in every rTMS patient, this would dramatically reduce the real-world utility of the technique - thus we would want to be very sure about how much benefit is really added by fMRI-guided targeting before proceeding to community implementation. I would suggest that it may be preferable to focus on comparing and optimizing protocols, while gathering neuroimaging data as a predictor of response at this point. This would circumvent potential problems with patients where the 'desired' connectivity pattern is either absent or reversed.
Does the plain English summary give a clear explanation of the research?<br><br>The summary gives a good account of the potential important role for rTMS in treatment-resistant depression, the rationale for the study, and the design and execution of the study. No changes to suggest.
Do you have any questions for the applicants that you would like the opportunity for the applicants to respond to prior to the proposal being considered by the funding board?<br><br>As above (see 'Weaknesses' section), it would be helpful for the authors to clarify:

Question and Response
1) how they would disambiguate the source of any superior efficacy in the cgiTBS group (imaging guidance versus theta burst protocol)<br><br>2) how they would handle cases in which the desired fMRI connectivity profile in the DLPFC is absent or reversed<br><br>It would also be helpful if the authors could clarify whether their pilot data are for the same protocol of 5 x 40 s of continuous theta-burst stimulation that they describe as their proposed intervention. This would normally be considered cTBS, not iTBS, and would normally be considered inhibitory, not excitatory. Although individuals respond heterogeneously to both cTBS and iTBS, cTBS has historically not performed particularly well (in either published work or our own experience) as a treatment for MDD, so the rationale for using it here rather than iTBS bears further explanation.

MRC Medical Research Council

NHS National Institute for Health Research

RESEARCH PROPOSAL EXTERNAL REVIEW OPEN COMMENTS

Review Questions

Question and Response
Issues for consideration by the board<br><br>The total amound of money requested appears high to me, but I am not familiar with research budgets and distribution of trial costs in the UK. Furthermore, costs are well motivated and include NHS care budget which is different from the situation in other countries. The number and background of staff requested to run the trial seems adequate.
What are the key strengths of this proposal?<br><br>- This is a clearly written and well motivated proposal to examine the clinical effects as well as the possible mechanism of action of cgiTBS versus rTMS in patients with moderate TRD (insufficient response to at least 2 antidepressants in current episode)<br>- The sample size is large and seems sufficient to deliver results that are relevant for patients.<br>- The applicants have done a pilot study that already showed a difference (not statistically significant) in favor of cgiTBS in a smaller group of patients.<br>- Patients and assessors are blind to treatment condition, this includes using a sham coil in all participants.<br>- The research network seems fit to conduct the trial and include the requested number of patients. This will however still be a challenge.
What are the key weaknesses of this proposal?<br><br>- I see no major weaknesses in this proposal.<br>- Both methods are believed to stimulate cortical function by affecting connectivity between relevant brain networks, probably through an increase in neuroplasticity. The mechanistic hypothesis is that anatomically guided cgiTBS shows larger effects than rTMS on the networks related to the insula with effects on cognition and symptomatology. The evidence fort his hypothesis does not seem to be very strong. Could it be that also rTMS focusing on a broader area might also, possibly as a secondary effect, influence the same networks?<br>- It is stated that side effects may be lower in cgiTBS than in rTMS because current is lower in the first, but I do the authors know literature describing such differences? Usually rTMS is also well tolerated.
Does the plain English summary give a clear explanation of the research?<br><br>Yes
Do you have any questions for the applicants that you would like the opportunity for the applicants to respond to prior to the proposal being considered by the funding board?<br><br>- I see no major weaknesses in this proposal.<br>- Both methods are believed to stimulate cortical function by affecting connectivity between relevant brain networks, probably through an increase in neuroplasticity. The mechanistic hypothesis is that anatomically guided cgiTBS shows larger effects than rTMS on the networks related to the insula with effects on cognition and symptomatology. The evidence fort his hypothesis does not seem to be very strong. Could it be that also rTMS focusing on a broader area might also, possibly as a

Question and Response

secondary effect, influence the same networks?

- It is stated that side effects may be lower in cgiTBS than in rTMS because current is lower in the first, but I do the authors know literature describing such differences? Usually rTMS is also well tolerated.

RESEARCH PROPOSAL EXTERNAL REVIEW OPEN COMMENTS

Review Questions

Question and Response

Issues for consideration by the board

The proposed research methods, recruitment and scientific quality appear to be robust and the recruitment process seems likely to work, given the input/pathways of referral including GPs, several well-established/experienced NHS hospitals/treatment centres, a trials unit and patient bodies/organisations.

A Clinical Trials Unit will be involved in the research management which seems a fantastic idea as they are likely to have the most expertise and experience when it comes to providing support for large multicentre randomised trials.

The research team appear to be adequately qualified, and salary costs for most clinicians/researchers are less than 10% FTE so is not financially burdensome and/or taking away too much money from frontline NHS staff costs. There appears to be an appropriate mix of skills between clinical staff, researchers and support staff.

The participant group is an important one as TRD is an area of unmet need and these patient suffer extensively, often not knowing exactly why or how long the condition will continue for. It is probably unlikely that many of the target group cannot work, so there should not be any major issues with attending trial visits/treatment sessions but they may need a carer to attend with them to provide support.

Adequate thought has also been given for a study website hosted at the Institute of Mental Health to provide information for all potential participants, referrers, researchers or other interested parties on the progress and results of the trial. This facilitates communication and information sharing/dissemination.

Overall the research methods seem appropriate and well thought out, particularly since the proposal was revised to effectively make it better and fulfil its objectives more comprehensively. The 4 main centres involved (Nottingham, Northampton, Newcastle and London) also cover different regions, as to capture a wide enough demographic/cross section of the target population, representative of this patient group across the UK.

I would probably be happy to become a participant, or for a friend or family member to become a participant in the research since the use of connectivity guided intermittent theta burst TMS sounds both interesting in the way it works and promising in terms of possible clinical benefit. Visit frequency is not too burdensome (baseline & then 8, 16 & 26 weeks from randomisation) but there needs to be sufficient support for patients between visits to ensure they have a point of contact and the proposal includes a safety plan to help those most at risk which is very encouraging.

It may be hard to engage with some TRD patients who may already be overly dependent on NHS services, or alternatively those who feel helpless/frustrated by lack of response to previous therapies and so are difficult to engage. A well run and professionally minded clinical trial could also improve engagement with patients, offering hope to this difficult to treat group who may not ordinarily engage

Question and Response
with health services i.e. they could feel encouraged to engage in a more consistent and meaningful way now as they feel encouraged that they could benefit clinically but also help to further science and medicine. It also offers an important solution to reduce the chronic burden of TRD and the psychosocial, financial, and long term wider economic problems associated with the condition.
What are the key strengths of this proposal?<br><br>MDD is a condition with high morbidity and mortality (risk of suicide) and may offer crucial benefits to patients who have not responded to other lines of NHS treatment.<br><br>Connectivity-based neuroimaging methods show promise of health benefit for TRD including in the Nottingham pilot study, so it's important to explore this further with a proper randomised trial, as in this proposal. The trial also explores whether outcomes are maintained at 16 weeks and 26 weeks, time points when standard NICE approved rTMS is not thought to be effective, so there is an opportunity to offer sustained benefit not currently provided by other modalities.<br><br>There are no multicentre efficacy RCTs of cgiTBS versus rTMS to date, and NICE has identied the need for further research which this trial helps to achieve. The investigation could be vital for improving TMS treatment in depression, which is a long term debilitating condition.<br><br>It is a non-invasive treatment so no pain, and side effects are likely to be minimal, if any. It is also multicentre, recruiting from primary and secondary care which could help to meet the significant recruitment target. Dissemination of information/aid to recruitment has also be considered by involving the Clinical Research Networks and service user organisations.<br><br>Qualitative interviews are a key strength to assess patient acceptability which is an important factor to consider when providing value for money in the NHS.<br><br>There has been substantial patient involvement in designing the trial (including the pilot study) in the form of an advisory group involving patients and carers.
What are the key weaknesses of this proposal?<br><br>Almost 1.8m total costs makes this a relatively expensive trial for the NHS with no guarantee of patient benefit but it is recognized that imaging-related treatments may naturally be costly compared to other forms of treatment for depression (e.g. medicines). NHS treatment costs do also appear to be comparatively low (less than £30,000).<br><br>No risk/benefit ratio or cost/quality of life comparison has been made between TBS and existing treatments effective for TRD (only mentioned ECT which may not be appealing due to side effects) but like-for-like treatment cost comparison would be helpful).<br><br>Preliminary data from the pilot RCT of 29 patients with TRD at Nottingham has shown significant improvement with cgiTBS in clinical response in depression symptoms but there doesn't appear to be any preliminary patient satisfaction/quality of life data from the pilot study. If not, why not? (may wish to explain reasons why it may not have been feasible).<br><br>No specific mention of whether the study can include pregnant/lactating women, or the elderly population (no defined upper age limit)
Does the plain English summary give a clear explanation of the research?

Question and Response
The first paragraph of the plain English summary is too long, so needs to be divided into 2/3 separate paragraphs.<br><br>Whilst all the required information is provided, some parts are unnecessarily wordy at times e.g. some patient might not really know what 'a pilot study' is, or using words like 'helped' instead of assisted would probably help. There are not too many highly scientific terms or repetitive abbreviations which is a positive point though.<br><br>Probably a good idea to get a PPI group to help further develop and even 'test out' the plain English summary on some real patients with depression before it is finalized and published. Diagrams would also be helpful to explain the science/brain biology.
Do you have any questions for the applicants that you would like the opportunity for the applicants to respond to prior to the proposal being considered by the funding board?<br><br>Treatment cost comparison (on a per patient basis over several weeks/months/years) versus well established (though obviously imperfect) alternative treatments for TRD such as antidepressants/ECT?<br><br>Are these alternatives a lot cheaper but of comparable efficacy? Can you provide historical data to compare with pilot study finding for TBS?<br><br>What are likely relapse rates for this novel treatment? Does the novel treatment reduce risk of relapse? If yes, how valuable is this benefit (both monetarily and in terms of reduced disease burden/improved patient quality of life (e.g. less time off work = potential value to the wider economy and society)<br><br>Does this novel treatment truly offer significant enough clinical benefit to justify trial costs and less value-for-money for patients/society (taxpayers)?

Applicant Response To External Review

Applicant Response

Reply to referees.

We have confined our replies to those issues that have been specifically raised for us to address in our comments.

1. Design of the trial – is it the neuroimaging guided TMS or the TBS associated with differences in response rates should these be demonstrated?

In response we would like to make the following points.

i. The issue with standard rTMS is that response occurs in just 25% of patients and this is sustained for just 3 to 4 weeks (Health Quality Ontario, 2016). In both our view as clinicians running treatment resistant depression (TRD) services and that of our PPI representatives, this severely limits the utility of standard rTMS in routine clinical practice. TRD is frequently chronic and/or highly recurrent severe mental illness.

ii. There is robust evidence base from meta-analyses that standard rTMS is more effective than placebo (NICE, 2015; Health Quality Ontario, 2016). However there is little of such evidence for other forms of TMS. We are comparing image guided TBS with rTMS on the basis of evidence suggesting that this is more effective. We are not comparing cgiTBS with sham treatment because a) the robustness of the data regarding rTMS provides us with a well characterised comparator, and b) the concerns of using a sham treatment in patients with severe treatment resistant illness delaying alternative treatment options. This latter issue was raised by the EME board at the outline stage and we adjusted the protocol accordingly.

iii. To date, meta-analysis has shown no convincing effects of any form of delivery of TMS over any other (Health Quality Ontario, 2016). A large Canadian RCT in around 400 patients with depression will show whether iTBS without connectivity guidance has a greater depression response and longer depression response than 10 Hz rTMS given over the DMPFC (Blumberger et al, 2016). However, there was no evidence of greater effectiveness in a non-randomised comparison of 185 patients given iTBS versus rTMS (Bakker et al, 2015). Our study will provide early confirmation (or otherwise) of the findings which come from Canada, but through the image guidance our study would substantially enhance the work through a clearer understanding of mechanisms. Moreover our pilot study demonstrated that connectivity guided TBS showed dramatically increased responses rates (88%) on two different measures of depression at 3 months, twice that of rTMS. This clinical response data is also backed by improvements in functional connectivity and GABA that are consistent with the proposed mechanisms of action reported in the literature. Connectivity guided iTBS was also associated with increasing the duration of response from 3 to 6 months, potentially enabling a considerable reduction in the need for other treatments, reducing both side-effects and costs. Consequently, a design comparing cgiTBS versus standard rTMS offers much better value because it offers a potential greater step change in treatment and provides more direct evidence of the mechanism of action of TBS.

Applicant Response

iv. TBS, and to a lesser extent all TMS, can induce both inhibitory and excitatory neurotransmission which if not neuroanatomically targeted may cancel each out (Huang et al, 2005). Our research in 27 volunteers (Iwabuchi et al, 2016) and our RCT data show sustained changes in functional connectivity and GABA were achieved by our TBS protocol (and to a greater extent than with rTMS) when the site of stimulation was anatomically localised close to the point of maximal effective functional connectivity of anterior insula to left DLPFC. Furthermore in patients with TRD changes in FC of the left DLPFC and DMPFC as well as GABA changes correlated inversely with changes in depression symptoms in both the TBS and rTMS groups. Our data provides preliminary evidence that connectivity guidance using neuroimaging improves the performance of rTMS and particularly iTBS. Therefore, we strongly disagree with the referee that our proposed RCT will slow progress of TMS research. If there is evidence of a much better response with cgiTBS compared to standard rTMS, this will be of great significance for a serious and costly disorder such as TRD and is likely to justify the use of neuroimaging.

v. We agree with the referee that iTBS shows some evidence of a longer duration of effect in depression response both in other RCTs and in our pilot RCT. However, the connectivity guided TBS we propose to use in our RCT is the same as in our pilot and is best characterised as iTBS, i.e short bursts of pulse delivery in combination with long periods of silent intervals. We administered iTBS, alternating 2 sec ON with 8 sec OFF. During the 2 second ON period we administered 10 triplets (3 cycles of 50Hz) at a rate of one triplet every 200ms, a total of 600 pulses. The above cycle was repeated 5 times with 5 minutes break following each cycle so there is a total of 3000 pulses per treatment. We abbreviated this description to save space.

vi. Resting fMRI (required to measure functional connectivity) using 3T scanners is now available at many NHS MRI units and the number of MRI units capable of doing this type of scanning is growing all the time due to its clinical utility for presurgical planning in brain tumours and in a range of neurological disorders e.g. migraine.. It is likely to soon be a standard technique offered throughout the NHS. rTMS is currently administered to some patients 3-4 times per year due to the lack of long term response. However, if cgiTBS gives a longer duration of response, as predicted, it may only need to be administered 1-2 times per year. This will help reduce the need for other treatments reducing costs and side effect burdens on patients. Additionally, the single structural and functional MRI scans needed prior to the first course however would not needed to be repeated for new courses of cgiTBS since the original data would be able to be used. Therefore, while undertaking cgiTBS would be slightly more complex than rTMS without neuroimaging, it may well be worth it in terms of clinical outcome, patient experience and economic outcomes.

vii. Although our design does not provide unambiguous identification of any advantage of iTBS separately from that of image guidance, we will perform secondary analyses to assess the possible gains from image guidance.: (i) We will record the number of participants who had the site of stimulation close the traditional site of stimulation at the DLPFC and hence would unlikely have benefited from the individual targeting. (ii) In the rTMS cohort we can compare the clinical response achieved without imaging guidance with the distance of the retrospectively determined connectivity based individual target site to test the hypothesis of lower response rate with increasing deviation of the actual target vs. the optimal target. (iii) We will determine the correlation between clinical response and eFC between insula and the actual site employed in the image guided iTBS arm. We will also use the rsFMRI data to estimate retrospectively the strength of eFC between the insula and the fixed standard DLPFC site. Comparison of the estimated eFC between insula and the standard site with the strength of eFC between the insula and the actual site of stimulation would provide an indication of the possible loss of clinical efficacy if stimulation had been applied at the standard site.

Applicant Response
viii. In summary, the RCT we propose of connectivity guided TBS versus standard rTMS represents a much greater advance in knowledge, will potentially lead to faster implementation of important therapeutic gains for patients with a severe life threatening long-term condition (TRD), and provide better value for money, than a study of TBS without connectivity guidance versus rTMS. By the time the RCT is completed resting fMRI required to guide the TBS will be almost universally available in the NHS. Our study will confirm or disconfirm the large Canadian RCT and will provide much more information on mechanism of action as well as test connectivity guided TBS where we already have evidence, both experimentally and theoretically, that it is likely to be superior to standard rTMS.<br><br>2. How will we handle the subset of connectivity patterns that have positive connectivity rather than negative connectivity in relation to the DLPFC or have the nearest site in the frontal lobe is well outside the DLPFC?<br><br>We agree that there are many patients with atypical functional connectivity patterns with sites of stimulation very far from the standard site of TBS or rTMS stimulation in the DLPFC. That is exactly why there is a need to accurately localise the target site for iTBS. For both the pilot and the proposed RCT, we will use a principled but pragmatic approach for site selection. Importantly, we based the selection on effective connectivity analysis (eFC) rather than simple functional connectivity based on simultaneous correlation between sites that the referee is referring to. Effective connectivity is computed using Granger Causality Analysis (GCA) and allows inference of the causal influence of activity in one brain region on another. In our opinion this approach is critical (and our pilot results provide preliminary evidence for this theoretical assumption) to the intended therapeutic effect of iTBS to altering the rAI network from a remote stimulation site. In order to be effective, the stimulated site has to be connected to the remote site and to be able to causally affect the remote site. To avoid the stimulation site to be outside of the DLPFC, we will constrain our GCA to an anatomical template as in the pilot study. While this approach worked reliably in our pilot study, should we encounter an unexpected (based on our pilot experience) case where we were unable to identify a clear eFC based DLPFC target, the DLPFC would be determined using individual functional connectivity using seed-based and independent component analysis to reconstruct the central executive network to define the DLPFC.<br><br>3. Contingency plans for other centres.<br><br>We are happy to provide a letter of support from the Birmingham site and have confirmation that this can be provided by e-mail if required. We have not arranged any contingencies with other sites. However, we would be happy to approach colleagues at the Institute of Psychiatry, South London (Prof Allan Young) should recruitment be slow in North London. The lead organisation already has a memorandum of understanding with South London to work on projects jointly between the Nottingham Biomedical Research centre mental health and technology theme in which Richard Morriss, Peter Liddle and Dorothee Auer are lead investigators for neuromodulation work in depression theme and the Maudsley Biomedical Research Centre which also has a number of mental health and technology themes. South London has the TMS, TRD service and neuroimaging facilities required for this study. We did not include South London as a centre in this proposal because they were leading on a rival bid in the same EME call. In both Birmingham and South London, the study sites could be supported by both research staff funded by the grant and CRN staff.

Applicant Response
4. Finance<br><br>Please explain further the need for 45 minutes for the TBS intervention and 20 minutes for the rTMS intervention listed under research costs in addition to the costs listed under NHS treatment costs.<br><br>The need for this additional time as a research cost relates to time taken for additional tasks because of the blinding of the participants and research assessors required in the RCT. TBS takes only 20 minutes to deliver while rTMS takes 45 minutes. In order to mask research assessors and participants properly, the duration of the treatment session, sound of the TMS, skin sensation of the TMS and the process of localising the TMS stimulation using MRI on the surface of the skull, must seem exactly the same. Sham TMS stimulation through a sham coil that sounds and feels like rTMS has to be delivered to the TBS group for 45 minutes only for blinding so this must be considered a research cost rather than a treatment cost. On the other hand both groups have to have the neuroanatomical guidance procedure – this is necessary to direct treatment in the TBS group but only necessary as a sham for research purposes in the rTMS group. So the additional 20 minutes in the rTMS group is also a research cost rather than a treatment cost. Therefore both groups will attend for 65 minutes but in the rTMS group, 20 minutes of this is for research reasons only and unnecessary in NHS practice; in contrast the TBS group would only need to be present for 20 minutes to receive their TBS with the rest of the 45 minutes, unnecessary for clinical practice, being taken up by blinding for research reasons, and is therefore also a research cost.<br><br>5. Intellectual Property<br><br>Please provide further information on who will own each element of IP such as the data, know-how and user feedback developed as part of the study. If this will not be the Trust, please explain why and please note clause 16.5 of the NIHR contract.<br><br>Data from the study would be owned by the party generating, in full compliance with applicable legislation. With rights to access to enable further research and teaching specified within the collaboration agreement, personal data and sensitive personal data within the meaning of the Data Protection Act will be dealt with as Confidential Information as specified within the NIHR contract. Foreground IP (including know-how) will be owned by Nottinghamshire Healthcare NHS Foundation Trust in line with NIHR policy, with all parties involved in the development of the IP being appropriately recognised. As detailed in the application, the University of Nottingham Technology Transfer Office and Nottinghamshire Healthcare NHS Foundation Trust have an established relationship regarding IP management. Prior written consent will be obtained from the NIHR should any commercial use Foreground IP be exploited as per clause 16.5 of the NIHR contract. If NIHR is using the term user feedback to mean some form of questionnaire/scale/tool then this would be owned by Nottinghamshire Healthcare NHS Foundation Trust along with the other Foreground IP.
