# Supplementary material for: Connectivity-Guided Theta Burst Transcranial Magnetic Stimulation Versus Repetitive Transcranial Magnetic Stimulation for Treatment-Resistant Moderate to Severe Depression: Magnetic Resonance Imaging Protocol and SARS-CoV-2–Induced Changes for a Randomized Double-blind Controlled Trial
Source: JMIR Res Protoc. 2022 Jan 20;11(1):e31925. doi: 10.2196/31925 (PMC8814922; doi:10.2196/31925)
Supplement: Multimedia Appendix 4 [file resprot_v11i1e31925_app4.pdf]

RESEARCH PROPOSAL EXTERNAL REVIEW OPEN COMMENTS

Review Questions

Question and Response
Issues for consideration by the board<br><br>This team have considered previous feedback from the board and redesigned parts of their study to take into account those issues raised. As a result the proposal is strong and well considered. It is underpinned by an experienced team who have piloted, and assessed the feasibility of both the randomised trial and the mechanistic sub-study thoroughly.
What are the key strengths of this proposal?<br><br>Excellent team with a strong background in the technology.<br>An infrastructure well equip for delivery of both the treatment and the research.<br>Good pilot and feasibility work in preparation.<br>Good links to the CTU.<br>Well worked power calculations and appropriate analysis plan.<br>Well powered and competently designed main RCT (I do not have sufficient knowledge of the mechanistic study to pass comment).<br>The inclusion of a short piece of qualitative work on user experience to help evaluate the utility of either arm in the case where both treatments were judged to be equally effective was a welcome addition.<br>The research costs are reasonable (total per participant costs of less than £5000 pp, with the mechanistic study yielding interesting information from both arms).
What are the key weaknesses of this proposal?<br><br>The health economic element is not articulated well and feels like a tack-on, the analysis detail in the main protocol seemed a little too generic. This HE evaluation could be a very valuable source of information on which to base commissioning decisions so it would seem a shame to underplay its potential. In a similar vein, it would have been good to see mention of a small process evaluation around implementation.<br>There were no design or statistical weaknesses that I could detect although the treatment of missing data values could always do with more careful consideration in the final statistical analysis plan.
Does the plain English summary give a clear explanation of the research?<br><br>I understood the research from the plain English summary, but the average character count was 5 per word and the average sentence was 20+ words. It would be possible to improve its readability and comprehensibility prior to publication by a good sub-editor.<br>The contents gave a good explanation of the background and the RCT.
Do you have any questions for the applicants that you would like the opportunity for the applicants to respond to prior to the proposal being considered by the funding board?<br><br>I have no further questions for the applicants as their application was comprehensive and clearly written.

RESEARCH PROPOSAL EXTERNAL REVIEW OPEN COMMENTS

Review Questions

Question and Response
Issues for consideration by the board<br><br>I feel that if the minor clarifications mentioned (under weaknesses) are answered that the trial should be funded and supported. I think that a letter of clarification would be sufficient. I also feel that a letter from Birmingham stating the support and expertise that they can give to the trial would be helpful.
What are the key strengths of this proposal?<br><br>This is now a much stronger proposal and a lot of preparatory work and thought has been given to the study. The study will help determine whether or not there is an advantage over an existing recommended treatment by NICE, There appears to be a very clear selection process and the research group have established TRD referral networks and prior trial experience in this area. A pilot study has already been performed and this establishes feasibility of the study. The team now have four centres, with the option of a fifth, so risks to recruitment are reduced.<br><br>There is a clear methodology for TRD assessment, measures and study criteria. There are clear exclusion criteria, it is appropriate that this now includes GABA modulating medication.<br>DSM comorbidity has been considered in exclusion, but (as mentioned in weaknesses)a plan of secondary analyses of any "allowed" comorbidties should be considered. The study has very clear outcome measures and study treatment outcomes and clinical correlation with hypothesised network changes. The team will use well respected outcome measures that can be directly compared with other data sets and discussion of inter-site training has been mentioned. Standardised self-rated measures are used for other outcomes.<br><br>Recruitment from secondary and primary care is pragmatic and used for feasibility reasons, I presume. Still it would be helpful to clarify whether any cohort differences are expected, or outcome differences. How many for example have had previous secondary care contact but have disengaged and are now managed in primary care?<br><br>Use of the Leicester site for trail co-ordination, established CRN links and preparatory work for the trial. Trial governance seems well thought out - but after the recruitment phase and at later points of the study, what would lead to trial stopping or redesign of protocol or SOPS? There is very well evidenced and advanced public and patient involvement and experience of this from previous trials.<br><br>Costs are very well justified and this is in real terms a relatively inexpensive trial and represents good value for investment.<br><br>The team is very well-balanced and have the required wide range of skills and experience. They have a history of successful collaboration and recruitment and of high quality published outputs.
What are the key weaknesses of this proposal?<br><br>This is a greatly strengthened proposal. Rather than definite weaknesses there are a few issues that need greater clarification. In the revised protocol there is no sham group, as discussed by the applicants for ethical reasons. It would be helpful to have a more detailed justification of the scientific

Question and Response
and ethical reasoning behind this change in protocol with a further definitive statement why this is not necessary i.e. pilots or RCTs already published and extent of evidence base.<br><br>In recruitment, analysis of any possible selection biases between sites and inter-site differences in protocol adherence could be addressed by the Trial Steering Group, possibly the applicants could give greater assurance om whether this is a possibility or unlikely.<br>For the entry criteria, I take it that moderate severity is determined by the standard HAM-D cut-off criteria e.g. 15, 17,21 etc., but clarification is important, Will the randomisation process and analysis look at severity strata.<br><br>Study burden is still an issue for recruitment and retention, can any flexibility be brought in to the protocol and SOPS to aid retention or is it not possible?<br><br>Analysis of extraneous treatments e.g CBT, etc and of other possible comorbidity not exluded e.g. trauma, personality disorder should be discussed.
Does the plain English summary give a clear explanation of the research?<br><br>This is an excellent summary of a complex study. Great care has been taken to explain randomisation, what exactly will happen and potential benefits and risks of the study. The information should be readily accessible for lay groups and potential study entrants.<br>Use of Leicester site and CRN for trial<br>DSM comorbidity considered (but see above)<br>Standardised self-rated measures<br>Very good to have contingency of extra site in Birmingham<br>Keeping most medication (except GABA modulating) and<br>Team well-balanced.
Do you have any questions for the applicants that you would like the opportunity for the applicants to respond to prior to the proposal being considered by the funding board?<br><br>I only have one, which is if the Birmingham or London centres cannot be available for any reason at the proposed start of the trial, are there any other contingency plans for other centres?

RESEARCH PROPOSAL EXTERNAL REVIEW OPEN COMMENTS

Review Questions

Question and Response
Issues for consideration by the board<br><br>This is a potentially interesting study seeking to establish whether a novel form of rTMS, featuring MRI guidance and a briefer 'theta-burst' stimulation protocol, performs better than the standard US FDA-approved protocol in treating medication-resistant depression. The study has a commendably large recruitment target of 368 patients, which may (just) be feasible across 4 sites in the time allotted. Patient inclusion and exclusion criteria and outcome measures are reasonable and match standard practices for such a study fairly well. The mechanistic side of this proposal is quite strong and features interesting studies of how rTMS affects the network activity of the brain on functional MRI, as well as the neurochemistry of the brain on MRS. That said, there are a couple of features of the design (particularly the novel versus comparator intervention) which may contain critical flaws that would preclude meaningful interpretation of the findings.<br><br>Major issues:<br><br>- As noted in the 'Weaknesses' section, the critical design flaw in this study is that the cgiTBS intervention differs in two potentially important ways from the standard intervention: different protocol (theta-burst), and different stimulation site (fMRI-guided). As such, there is no way to disambiguate whether a superior effect for cgiTBS stems from higher potency of theta-burst stimulation or from more precise, individualized targeting of the stimulation site. The real-world impact of this is that one might falsely conclude that every patient presenting for rTMS will need to undergo fMRI before treatment in order to maximize the chances of success. If so, rTMS may have trouble making a meaningful reduction in the 2% of the population with treatment-resistant depression. The present study design therefore runs the risk of making it less clear, rather than more clear, whether rTMS is a value proposition for the public health system as a whole.<br><br>- A second major technical issue is that the MRI-guided stimulation site may, in some patients, be almost the same as the non-MRI-guided stimulation site; in other patients, the MRI-guided site could potentially be difficult to identify at all (as detailed below). Thus, it may be difficult to randomize patients in a meaningful way to MRI-guided versus non-MRI-guided stimulation arms.<br><br>Minor Issues:<br>- The authors describe pilot data for 'iTBS', which is normally considered an excitatory protocol and refers to a pattern of 2 s on, 8 s off x 20 trains of 50 Hz triplet pulses. However, their intervention protocol described 5 runs of a 40 s continuous train of 50 Hz triplet pulses - this would normally be considered 5 runs of continuous TBS or 'cTBS', generally considered an inhibitory protocol. cTBS, in published work and in our own experience, has not performed as well as iTBS. It would therefore be helpful to clarify if the authors' promising pilot work is from the same 5x cTBS protocol they are proposing to use here, or from 'true' iTBS as described above, as these two protocols are reckoned to have opposite effects. It would also be helpful to clarify why they appear to refer to their protocol as 'iTBS' when its parameters appear closer to cTBS repeated 5 times.
What are the key strengths of this proposal?<br><br>There are several encouraging strengths to this study:

Question and Response
- Theta-burst rTMS has the potential to dramatically improve the real-world utility of rTMS as a tool for making meaningful reductions in the 2% of the population with medication-resistant depression, at reasonable cost. As such, the potential impact could be high<br><br>- There are so far very few large-N studies to establish the relative efficacy of theta-burst stimulation over conventional longer protocols (although at least 1 large Canadian study of conventional 10 Hz vs short theta-burst rTMS is near completion, and will likely be published several years before this study is complete).<br><br>- The use of neuroimaging for individualized guidance is a 'gold-standard' approach for precision (although if the treatment truly proves to require MRI in every patient, its real-world impact may be reduced significantly)<br><br>- The enrolment of 368 patients would position the study as one of the largest brain-stimulation studies yet conducted (although a recruitment period of just 25 months is rather ambitious for such a sample in a 4-site study and would require sustained recruitment rates of ~1 patient per week per site - not unheard of, but worth providing supportive records to demonstrate feasibility)<br><br>- The team appears to have good experience in brain stimulation, depression, and neuroimaging<br><br>- Patient recruitment plans, inclusion and exclusion criteria, and outcome measures appear reasonable (although the intervention and comparator aspects of the design may have a critical flaw, as below)<br><br>- The collected data (functional MRI and MRS) in such a large patient sample would be a rich source of insight into the mechanisms of rTMS at the neurophysiological level<br><br>- The authors' hypotheses regarding DLPFC-DMPFC connectivity, DLPFC-insula connectivity, and prefrontal GABA (on MRS spectra) as mechanisms of action are intriguing and backed by interesting pilot data<br><br>- Economic and quality-of-life analyses are an important adjunct to the efficacy and mechanistic objectives of the proposal
What are the key weaknesses of this proposal?<br><br>There are some problematic features with both the strategy and the design of the study as presently proposed:<br><br>- The most critical design issue is that the novel intervention (cgiTBS) differs from the standard comparator intervention (10 Hz rTMS) in more than one way that is hypothesized to affect efficacy. Not only is the stimulation protocol different (theta-burst rather than standard 10 Hz), but the stimulation site is also different (fixed F5 location versus functional MRI-guided location). In the event that the study shows superiority for cgiTBS over conventional stimulation, there is no straightforward way to determine whether the improved outcomes arise from the theta-burst pattern of stimulation, or the more individualized targeting of the stimulation site, or some combination of the two.<br><br>- This issue has the potential to diminish the real-world impact of the study: for example, if the benefits are actually from the theta-burst stimulation and not from the fMRI-guided fine-tuning of the

**Question and Response**
target, one might falsely conclude that all patients need to undergo a costly and limited-access fMRI scan in order for rTMS to work properly.<br><br>- The requirement for a functional MRI session in every patient would markedly reduce the practicality of rTMS as a strategy for making meaningful reductions in the 2% of the population with treatment-resistant depression, so we would want very clear evidence on exactly how much benefit is conferred by fine-tuning the target using rTMS; the present study design does not enable us to determine whether any added benefits are from the fMRI guidance versus simply the theta-burst pattern of stimulation.<br><br>- There are also two practical problems with randomizing patients to fMRI-guided targeting versus a standard location:<br><br>First, for a substantial proportion of patients, in our experience, the fMRI-guided target ends up being less than 5mm from the fixed target in any case, so randomizing them to fMRI guidance ends up giving them more or less the same site as they would have had if they had been randomized to the fixed target (since the inter-session and inter-operator errors are usually on the order of 4-8 mm in any case). I do not see an easy remedy for this issue aside from enrolling a very large number of patients, treating them all according to some standard scalp heuristic generating high variance of sites across individuals, and then comparing outcomes as a function of the rTMS coil's distance from the fMRI-guided 'ideal' target in each person, having controlled for the many other variables that might also affect outcome. Logistically this would be rather tricky.<br><br>Second, for a subset of patients (up to 30% in our experience), connectivity patterns to the DLPFC have the opposite sign to the typical patient (e.g., positive connectivity rather than negative connectivity), or the nearest site in the frontal lobe with the desired connectivity sign ends up being well outside the DLPFC. It is not clear how the applicants propose to handle cases of patients who have the 'wrong' sign of connectivity in the target region, or whose connectivity target lies outside the DLPFC as typically defined.<br><br>In summary, although the successful use of neuroimaging for individualized rTMS targeting is a 'holy grail' for the field, and although theta-burst stimulation is a very promising innovation among rTMS protocols, the incorporation of both of these features simultaneously into the randomization is a critical conceptual flaw in the design. Moreover, if neuroimaging actually does turn out to be required in every rTMS patient, this would dramatically reduce the real-world utility of the technique - thus we would want to be very sure about how much benefit is really added by fMRI-guided targeting before proceeding to community implementation. I would suggest that it may be preferable to focus on comparing and optimizing protocols, while gathering neuroimaging data as a predictor of response at this point. This would circumvent potential problems with patients where the 'desired' connectivity pattern is either absent or reversed.
Does the plain English summary give a clear explanation of the research?<br><br>The summary gives a good account of the potential important role for rTMS in treatment-resistant depression, the rationale for the study, and the design and execution of the study. No changes to suggest.
Do you have any questions for the applicants that you would like the opportunity for the applicants to respond to prior to the proposal being considered by the funding board?<br><br>As above (see 'Weaknesses' section), it would be helpful for the authors to clarify:

Question and Response
1) how they would disambiguate the source of any superior efficacy in the cgiTBS group (imaging guidance versus theta burst protocol)<br><br>2) how they would handle cases in which the desired fMRI connectivity profile in the DLPFC is absent or reversed<br><br>It would also be helpful if the authors could clarify whether their pilot data are for the same protocol of 5 x 40 s of continuous theta-burst stimulation that they describe as their proposed intervention. This would normally be considered cTBS, not iTBS, and would normally be considered inhibitory, not excitatory. Although individuals respond heterogeneously to both cTBS and iTBS, cTBS has historically not performed particularly well (in either published work or our own experience) as a treatment for MDD, so the rationale for using it here rather than iTBS bears further explanation.

RESEARCH PROPOSAL EXTERNAL REVIEW OPEN COMMENTS

Review Questions

Question and Response
Issues for consideration by the board<br><br>The total amound of money requested appears high to me, but I am not familiar with research budgets and distribution of trial costs in the UK. Furthermore, costs are well motivated and include NHS care budget which is different from the situation in other countries. The number and background of staff requested to run the trial seems adequate.
What are the key strengths of this proposal?<br><br>- This is a clearly written and well motivated proposal to examine the clinical effects as well as the possible mechanism of action of cgiTBS versus rTMS in patients with moderate TRD (insufficient response to at least 2 antidepressants in current episode)<br>- The sample size is large and seems sufficient to deliver results that are relevant for patients.<br>- The applicants have done a pilot study that already showed a difference (not statistically significant) in favor of cgiTBS in a smaller group of patients.<br>- Patients and assessors are blind to treatment condition, this includes using a sham coil in all participants.<br>- The research network seems fit to conduct the trial and include the requested number of patients. This will however still be a challenge.
What are the key weaknesses of this proposal?<br><br>- I see no major weaknesses in this proposal.<br>- Both methods are believed to stimulate cortical function by affecting connectivity between relevant brain networks, probably through an increase in neuroplasticity. The mechanistic hypothesis is that anatomically guided cgiTBS shows larger effects than rTMS on the networks related to the insula with effects on cognition and symptomatology. The evidence fort his hypothesis does not seem to be very strong. Could it be that also rTMS focusing on a broader area might also, possibly as a secondary effect, influence the same networks?<br>- It is stated that side effects may be lower in cgiTBS than in rTMS because current is lower in the first, but I do the authors know literature describing such differences? Usually rTMS is also well tolerated.
Does the plain English summary give a clear explanation of the research?<br><br>Yes
Do you have any questions for the applicants that you would like the opportunity for the applicants to respond to prior to the proposal being considered by the funding board?<br><br>- I see no major weaknesses in this proposal.<br>- Both methods are believed to stimulate cortical function by affecting connectivity between relevant brain networks, probably through an increase in neuroplasticity. The mechanistic hypothesis is that anatomically guided cgiTBS shows larger effects than rTMS on the networks related to the insula with effects on cognition and symptomatology. The evidence fort his hypothesis does not seem to be very strong. Could it be that also rTMS focusing on a broader area might also, possibly as a

Question and Response
secondary effect, influence the same networks?<br>- It is stated that side effects may be lower in cgiTBS than in rTMS because current is lower in the first, but I do the authors know literature describing such differences? Usually rTMS is also well tolerated.

RESEARCH PROPOSAL EXTERNAL REVIEW OPEN COMMENTS

Review Questions

Question and Response

Issues for consideration by the board

The proposed research methods, recruitment and scientific quality appear to be robust and the recruitment process seems likely to work, given the input/pathways of referral including GPs, several well-established/experienced NHS hospitals/treatment centres, a trials unit and patient bodies/organisations.

A Clinical Trials Unit will be involved in the research management which seems a fantastic idea as they are likely to have the most expertise and experience when it comes to providing support for large multicentre randomised trials.

The research team appear to be adequately qualified, and salary costs for most clinicians/researchers are less than 10% FTE so is not financially burdensome and/or taking away too much money from frontline NHS staff costs. There appears to be an appropriate mix of skills between clinical staff, researchers and support staff.

The participant group is an important one as TRD is an area of unmet need and these patient suffer extensively, often not knowing exactly why or how long the condition will continue for. It is probably unlikely that many of the target group cannot work, so there should not be any major issues with attending trial visits/treatment sessions but they may need a carer to attend with them to provide support.

Adequate thought has also been given for a study website hosted at the Institute of Mental Health to provide information for all potential participants, referrers, researchers or other interested parties on the progress and results of the trial. This facilitates communication and information sharing/dissemination.

Overall the research methods seem appropriate and well thought out, particularly since the proposal was revised to effectively make it better and fulfil its objectives more comprehensively. The 4 main centres involved (Nottingham, Northampton, Newcastle and London) also cover different regions, as to capture a wide enough demographic/cross section of the target population, representative of this patient group across the UK.

I would probably be happy to become a participant, or for a friend or family member to become a participant in the research since the use of connectivity guided intermittent theta burst TMS sounds both interesting in the way it works and promising in terms of possible clinical benefit. Visit frequency is not too burdensome (baseline & then 8, 16 & 26 weeks from randomisation) but there needs to be sufficient support for patients between visits to ensure they have a point of contact and the proposal includes a safety plan to help those most at risk which is very encouraging.

It may be hard to engage with some TRD patients who may already be overly dependent on NHS services, or alternatively those who feel helpless/frustrated by lack of response to previous therapies and so are difficult to engage. A well run and professionally minded clinical trial could also improve engagement with patients, offering hope to this difficult to treat group who may not ordinarily engage

Question and Response
with health services i.e. they could feel encouraged to engage in a more consistent and meaningful way now as they feel encouraged that they could benefit clinically but also help to further science and medicine. It also offers an important solution to reduce the chronic burden of TRD and the psychosocial, financial, and long term wider economic problems associated with the condition.
What are the key strengths of this proposal?<br><br>MDD is a condition with high morbidity and mortality (risk of suicide) and may offer crucial benefits to patients who have not responded to other lines of NHS treatment.<br><br>Connectivity-based neuroimaging methods show promise of health benefit for TRD including in the Nottingham pilot study, so it's important to explore this further with a proper randomised trial, as in this proposal. The trial also explores whether outcomes are maintained at 16 weeks and 26 weeks, time points when standard NICE approved rTMS is not thought to be effective, so there is an opportunity to offer sustained benefit not currently provided by other modalities.<br><br>There are no multicentre efficacy RCTs of cgiTBS versus rTMS to date, and NICE has identied the need for further research which this trial helps to achieve. The investigation could be vital for improving TMS treatment in depression, which is a long term debilitating condition.<br><br>It is a non-invasive treatment so no pain, and side effects are likely to be minimal, if any. It is also multicentre, recruiting from primary and secondary care which could help to meet the significant recruitment target. Dissemination of information/aid to recruitment has also be considered by involving the Clinical Research Networks and service user organisations.<br><br>Qualitative interviews are a key strength to assess patient acceptability which is an important factor to consider when providing value for money in the NHS.<br><br>There has been substantial patient involvement in designing the trial (including the pilot study) in the form of an advisory group involving patients and carers.
What are the key weaknesses of this proposal?<br><br>Almost 1.8m total costs makes this a relatively expensive trial for the NHS with no guarantee of patient benefit but it is recognized that imaging-related treatments may naturally be costly compared to other forms of treatment for depression (e.g. medicines). NHS treatment costs do also appear to be comparatively low (less than £30,000).<br><br>No risk/benefit ratio or cost/quality of life comparison has been made between TBS and existing treatments effective for TRD (only mentioned ECT which may not be appealing due to side effects) but like-for-like treatment cost comparison would be helpful).<br><br>Preliminary data from the pilot RCT of 29 patients with TRD at Nottingham has shown significant improvement with cgiTBS in clinical response in depression symptoms but there doesn't appear to be any preliminary patient satisfaction/quality of life data from the pilot study. If not, why not? (may wish to explain reasons why it may not have been feasible).<br><br>No specific mention of whether the study can include pregnant/lactating women, or the elderly population (no defined upper age limit)
Does the plain English summary give a clear explanation of the research?

Question and Response
The first paragraph of the plain English summary is too long, so needs to be divided into 2/3 separate paragraphs.<br><br>Whilst all the required information is provided, some parts are unnecessarily wordy at times e.g. some patient might not really know what 'a pilot study' is, or using words like 'helped' instead of assisted would probably help. There are not too many highly scientific terms or repetitive abbreviations which is a positive point though.<br><br>Probably a good idea to get a PPI group to help further develop and even 'test out' the plain English summary on some real patients with depression before it is finalized and published. Diagrams would also be helpful to explain the science/brain biology.
Do you have any questions for the applicants that you would like the opportunity for the applicants to respond to prior to the proposal being considered by the funding board?<br><br>Treatment cost comparison (on a per patient basis over several weeks/months/years) versus well established (though obviously imperfect) alternative treatments for TRD such as antidepressants/ECT?<br><br>Are these alternatives a lot cheaper but of comparable efficacy? Can you provide historical data to compare with pilot study finding for TBS?<br><br>What are likely relapse rates for this novel treatment? Does the novel treatment reduce risk of relapse? If yes, how valuable is this benefit (both monetarily and in terms of reduced disease burden/improved patient quality of life (e.g. less time off work = potential value to the wider economy and society)<br><br>Does this novel treatment truly offer significant enough clinical benefit to justify trial costs and less value-for-money for patients/society (taxpayers)?

Applicant Response To External Review

Applicant Response
Thank you for the opportunity to respond to two further reviewers.<br><br>1. Both iTBS and rTMS affect connectivity through increases in neuroplasticity.<br><br>We agree with the referee on the basis of our pilot data that both iTBS and rTMS stimulate cortical function by affecting connectivity between relevant brain networks. However, the effects of rTMS are broad and are not focussed on the optimal site of stimulation as well as connectivity guided iTBS. Moreover in other work carried out experimentally, TBS is likely to result in long-term potentiation proximally to the site of stimulation as well as more distally e.g. Huang 2005. Such long-term potentiation distal to the site of stimulation has not been clearly established with rTMS. Empirically in many RCTs the effect of rTMS on depression response lasts for 3-4 weeks and then starts to diminish. In our pilot, even with connectivity guidance, the depression response following rTMS had started to wane by 3 months whereas with iTBS it had actually increased at 3 months compared to 1 month. Overall connectivity guided iTBS is therefore likely to have a more sustained effect on the depression response in comparison with rTMS by longer term modulation of network connectivity and inhibitory (GABA) tone. We hypothesise that the more sustained depression response of iTBS is achieved by focussing the TBS stimulation on the prefrontal network hub most closely linked to the anterior insula. The proposed study will provide mechanistic evidence for this hypothesised mode of action but also allow us to test an alternative hypothesis related to direct modulation of activity of the nexus of the affective, cognitive control and default mode networks as well as the inherent property of TBS to produce long-term potentiation both proximally and distally within these networks.<br><br>2. Side-effects of TBS versus rTMS.<br><br>We propose to address these issues. We agree that rTMS is very well tolerated and side-effects tend to be mild and we expect iTBS to be similar. Hong et al (2015) reported that in a non-randomised study of 165 participants who completed a 5 point acceptability and side-effect checklist, only 10.5% iTBS had side-effects, all of which were mild except for one participant. In our pilot study, one participant withdrew from each of the iTBS and rTMS arms, one for syncope receiving rTMS and one because of competing time commitments but not side-effects from iTBS. The reason why we suspect iTBS may be more acceptable to patients is based on the reduced duration (iTBS 20 minutes versus rTMS 45 minutes) and strength of stimulation (iTBS 80% motor threshold versus 120% for rTMS). No study of TMS has reported qualitative data to explore patient acceptability as our PPI group have clearly requested. We will thoroughly test whether there are any differences in side-effects and tolerability between connectivity guided iTBS and rTMS empirically and systematically using both

**Applicant Response**
quantitative and qualitative methods with PPI input.<br><br>3. Cost comparison and efficacy of rTMS and TBS.<br><br>A number of RCTs and meta-analyses have shown that ECT is more effective but less well tolerated than standard rTMS for TRD resulting in ECT being only marginally more cost effective e.g. Knapp et al (2008). On the basis of one RCT, ECT was more cost effective than rTMS at NICE threshold of £20,000 per QALY (Vallejo-Torres et al, 2015) and ECT was more cost effective at willingness-to-pay at £25,800 (30,000 Euros) per QALY (Health Care Ontario, 2016) on the basis of meta-analysis of RCTs of ECT versus standard rTMS at £23,337 per QALY. These findings are in part driven by the relatively short period of sustained improvement following rTMS. If connectivity guidance iTBS provided a more sustained depression response, as our previous data suggests, iTBS should be more clinically and cost effective compared to ECT, as well as being safer and better tolerated without severe adverse effects such as memory loss.<br><br>Compared to standard antidepressant care for TRD, standard rTMS has been shown to be as effective and either produce net savings or better cost effectiveness. Simpson et al (2009) report a saving of £898 per QALY in direct health care costs and £6096 per QALY in society costs mainly because of gains in productivity and work. Nguyen and London (2015) found standard rTMS to be more cost effective than antidepressant treatment for TRD at a willingness-to –pay threshold of £30,000. Again connectivity guided-TBS should produce similar or greater savings and cost effectiveness.<br><br>Morriss et al (2016) compared a specialist depression service providing comprehensive psychotherapy and antidepressant treatment for TRD. Compared to standard antidepressant and psychotherapy in secondary care mental health services, the specialist depression service produced clinically important and significant improvements in depression symptoms over 18 months compared with standard care for people with persistent moderate to severe depression, but at twice the cost so overall the cost of comprehensive care as twice the NICE threshold of £20,000 per QALY.<br><br>Connectivity guided iTBS, if it could be repeated with only the need for fMRI and structural MRI on the first occasion, is likely to produced sustained clinical benefit. Whether this benefit is calculated as depression response or by QALY cgiTBS may show improved cost effectiveness at NICE thresholds for willingness-to-pay or even both improved outcome and reduced costs versus any alternative treatment for TRD (rTMS, ECT, antidepressant care or combined antidepressant and psychotherapy). If society costs are also included, as we propose in our health economics analysis, we expect even greater savings and improved cost effectiveness based on the analysis by Simpson et al (2009). These wider society savings are likely to include improved work productivity, benefit payments, parental and carer roles, reduced suicidality and mortality and social care from comorbid physical illness where TRD is known to worsen the prognosis.

**Applicant Response**
Bearing in mind that TRD may account for 75% of the cost of all the costs of depression as outlined already in the application, the costs of this RCT and mechanism study are more than outweighed by the benefits that may stem from it. All 5 reviewers agree that the study is feasible and likely to provide valuable evidence, and 4 out of 5 who believe the design is also optimal.<br><br>References.<br><br>Hong YH et al. Safety and tolerability of theta burst stimulation vs. single and paired pulse transcranial magnetic stimulation: a comparative study of 165 pediatric subjects. Front Hum Neurosci. 2015;9:29.<br><br>Knapp M et al. Cost-effectiveness of transcranial magnetic stimulation vs. electroconvulsive therapy for severe depression: a multi-centre randomised controlled trial. J Affect Disord. 2008;109:273-85.<br><br>Morriss R et al. Clinical and cost effectiveness of a specialist depression service versus usual specialist mental health care for managing persistent depression (CLAHRC): a RCT. Lancet Psychiatry.2016;3:821-31.<br><br>Nguyen KH, Gordon LG. Cost-Effectiveness of Repetitive Transcranial Magnetic Stimulation versus Antidepressant Therapy for Treatment-Resistant Depression. Value Health. 2015;18:597-604.<br><br>Health Quality Ontario. Repetitive Transcranial Magnetic Stimulation for Treatment-Resistant Depression: An Economic Analysis. Ont Health Technol Assess Ser. 2016;16:1-51.<br><br>Simpson KN et al. Cost-effectiveness of TMS in the treatment of major depression: a health economics analysis. Adv Ther 2009; 26: 346–368.<br><br>Vallejo-Torres L et al. Cost-effectiveness of electroconvulsive therapy compared to repetitive transcranial magnetic stimulation for treatment-resistant severe depression: a decision model.
